# Retinal astrocyte morphology predicts integration of vascular and neuronal architecture

**DOI:** 10.3389/fnins.2023.1244679

**Published:** 2023-08-09

**Authors:** Joseph M. Holden, Lauren K. Wareham, David J. Calkins

**Affiliations:** ^1^Department of Ophthalmology and Visual Sciences, Vanderbilt University Medical Center, Nashville, TN, United States; ^2^Vanderbilt Neuroscience Graduate Program, Vanderbilt University, Nashville, TN, United States

**Keywords:** retina, astrocyte, glia, morphology, neurovascular-unit, vasculature, ganglion cells

## Abstract

Astrocytes are important regulators of blood flow and play a key role in the response to injury and disease in the central nervous system (CNS). Despite having an understanding that structural changes to these cells have consequences for local neurovascular physiology, individual astrocyte morphology remains largely unexplored in the retina. Here, we used MORF3 mice to capture full membranous morphology for over fifteen hundred individual astrocytes in the mouse retina, a highly metabolically active component of the CNS. We demonstrate that retinal astrocytes have been misrepresented as stellate in morphology due to marker use like GFAP and S100β which underestimates cell complexity. We also find that astrocytes contain recurring morphological motifs which are predictive of the underlying neurovascular architecture of the inner retina and suggestive of function. These motifs predict fine sampling and integration of retinal ganglion cell electrical activity with consequences for blood flow regulation. Additionally, our data shows that astrocytes participate in neurovascular interactions to a much greater degree than currently reported. 100% of cells contact the vasculature through one of three mutually exclusive classes of connections. Similarly, 100% of cells contact some neuronal element, be it an RGC axon or soma. Finally, we report that astrocyte morphology depends on retinal eccentricity, with cells appearing compressed near the nerve head and in the periphery. These results reveal a large degree of astrocyte morphological complexity that informs their contribution to neurovascular coupling in the retina.

## Introduction

Astrocytes engage in a variety of functions integral to the health of the central nervous system (CNS). These include synapse and blood–brain barrier maintenance, neurotrophic support, and the regulation of blood flow (Takano et al., 2006; Liddelow and Barres, 2015; Newman, 2015; Colombo and Farina, 2016; Allen and Eroglu, 2017; Heithoff et al., 2021). In response to injury and disease, astrocyte function and morphology change concurrently (Sun et al., 2010; Gallego et al., 2012; Schiweck et al., 2018; Pekny et al., 2019). Morphological changes, such as overall cytoskeletal hypertrophy, are largely assessed as a population, rather than for individual cells and their microstructures (Chen et al., 1993; Wilhelmsson et al., 2006). Historically, it has been difficult to label individual astrocytes for full-membranous morphology while distinguishing clear boundaries between adjacent cells. Because astrocytes form an extensive network, labeling cytoskeletal or cytoplasmic proteins is not sufficient to resolve single-cell morphology (Hösli et al., 2022). Approaches like single-cell dye filling, which do allow such resolution, are very low throughput and cannot easily reveal changes which occur in disease or injury. These ambiguities obscure clear understanding of astrocyte structure and function.

Morphological changes in retinal astrocytes are associated with highly prevalent degenerative diseases, including glaucomatous optic neuropathy and diabetic retinopathy (Ly et al., 2011; Lye-Barthel et al., 2013; Tham et al., 2014; Lee et al., 2015; García-Bermúdez et al., 2021; Shinozaki et al., 2023). Here, we quantify full membranous morphology of astrocytes in the mouse retina at single-cell resolution. To do so, we crossed two commercially available mouse strains: a glial-fibrillary acidic protein (GFAP) Cre line and the mononucleotide repeat frameshift (MORF3) mouse line (Gregorian et al., 2009; Veldman et al., 2020). Progeny from this cross are referred to as G-MORF mice. They exhibit sparse labeling of cells which have expressed GFAP at some point in development with a membrane-directed, highly antigenic fluorescent reporter. High-resolution confocal imaging of retinal astrocytes from naïve mice has revealed recurring structural motifs, which are present across all cells. These motifs act as building blocks, providing a simplifying order to describe much of the morphological heterogeneity in the retinal astrocyte population. Our results also reveal that every retinal astrocyte directly contacts at least one blood vessel and some neuronal element, be it an RGC axon or cell body. This work provides evidence for a more extensive degree of retinal astrocyte-neurovascular unit (NVU) participation than is currently reported in the literature. These data will serve as a baseline from which comparisons can be made using injury and disease models.

## Methods

### Animals

All animals used in this study were adult mice (8–12 weeks) on a C57 Black 6 background, with equal numbers of males and females. Animals were housed at the Vanderbilt University Division of Animal Care facility and subjected to a 12-h light/dark cycle. Animals were provided with water and rodent chow *ad libitum*. Cre 77.6 mice (Jackson Labs #024098) were crossed with MORF3 mice (Jackson Labs #035403) to generate G-MORF mice. In G-MORF mice, a highly antigenic spaghetti monster fluorescent protein (smFP) with 20 V5 epitope tags is targeted to cell membranes through a farnesylation sequence (Hancock et al., 1991). Critically, this construct requires a stochastic frameshift mutation during development for the smFP to be properly translated. This feature results in around 1% of total retinal astrocyte labeling with smFP, enabling individual cells to be distinguished from one another (Supplementary Figure S1). Each mouse yields between 75 and 150 well-separated, membrane-labeled astrocytes per retina allowing for morphology visualization at a quality comparable to whole-cell dye filling (Supplementary Figures S1, S3). The smFP is not endogenously fluorescent. For whole-cell patch dye filling, we crossed Cre 77.6 mice with a floxed tdTomato reporter mouse (Jackson Labs #007914).

One week prior to sacrifice, mice were anesthetized with a solution of ketamine / xylazine (90 mg/kg ketamine, 5 mg/kg xylazine) and injected with a solution of 1% fluorescein in 1X PBS. An angiogram was then taken as a retinal fingerprint which would later allow for the determination of retinal orientation following immunohistochemistry. G-MORF mice were sacrificed by means of intraperitoneal pentobarbital injection followed by transcardial perfusion of 1X PBS. Eyes were immediately enucleated, and the retinas dissected fresh. Retinas were transferred to a solution of collagenase (LS005273, Worthington Biochemical) and hyaluronidase (LS002592, Worthington Biochemical) in Ame’s media (A1372-25, US Biologic) for 10 min at room temperature to digest the vitreous. Following this incubation, vitreous was removed as a single sheet using forceps. Relief cuts were made to divide the tissue into quadrants and the retinas then transferred to a solution of 4% paraformaldehyde to shake at room temperature for 1 h. Retinas were stored in 1X PBS with azide at 4°C until used.

### Immunohistochemistry

Tissue was blocked in a solution of 5% normal donkey serum (NDS) in 0.1% Triton-X for 3 h, while shaking at room temperature. The tissue was then incubated in a solution of primary antibody in 0.1% Triton-X with 3% NDS for 3 days, while shaking at 4°C. Primary antibodies used in this paper include: rabbit anti-V5 (1:500, Bethyl Laboratories, A190-120A), goat anti-V5 (1,500, Abcam, ab95038), isolectin GS-IB4 biotin-XX conjugate (1,500; Invitrogen, I21414), goat anti-GFAP (1,500, Abcam, ab53554), rabbit anti-NG2 (1,500, Millipore, AB5320), mouse anti-SMI-31 (1,500, Biolegend, 801,601), rabbit anti-NLRP3 (1,250, Cell Signaling, 15,101), goat anti-IBA1 (1,250, Novus Biologicals, NB100-1028), and mouse anti-beta III tubulin (1,500, Millipore, MAB5564). Following three five-minute washes of the retinas in 1X PBS, tissue was incubated in a solution of secondary antibody. Secondary antibodies and probes used in this paper include donkey anti-rabbit conjugated to Alexa 488 (1,200, Jackson ImmunoResearch, 711–545-152), donkey anti-goat conjugated to Alexa 488 (1,200, Jackson ImmunoResearch, 705–545-003), donkey anti-rabbit conjugated to Alexa 555 (1,200, Invitrogen, A-31572), donkey anti-goat conjugated to Cy3 (1,200, Jackson ImmunoResearch, 705–165-147), streptavidin conjugated to Alexa 647 (1,200, Invitrogen, S21374), and phalloidin conjugated to Alexa 647 (1,200, Invitrogen, A22287). Retinae were washed three times for 5 minutes with 1X PBS and then mounted with Fluoromount-G on slides for imaging.

### Whole-cell dye filling

Animals were euthanized by cervical dislocation and eyes dissected under red light conditions (630 nm, 800 μW/cm2, FND/FG, Ushio, Cypress, CA). Retinas were transferred to a carbogen-saturated Ames’ medium (US Biologic, Memphis, TN) supplemented with 20 mM D-glucose and 22.6 mM NaHCO3 (pH 7.4, 294 Osm). Relief cuts were made into each retina and flat mounted onto a physiological chamber. Ames’ media perfused each retina at a flow rate of 2 mL/min at 35°C (Model TC-344C, Warner Instruments, Hamden, CT). Individual astrocytes were located using a 40X water immersion objective on an Olympus BX50 microscope. Cells were patched onto in a whole-cell configuration using a borosilicate pipette (I.D. 0.86 mm, O.D. 1.5 mm; Sutter Instruments, Novato, CA) filled with (in mM): 125 K-gluconate, 10 KCl, 10 HEPES, 10 EGTA, 4 Mg-ATP, 1 Na-GTP, 0.1 Alexa 488 and 0.1 Alexa 647 dye (Invitrogen, Carlsbad, CA). The intracellular solution pH was 7.35 and osmolarity was 285 Osm.

### Imaging

Immunolabeled retinas were first imaged at 20X magnification on a Nikon Ti-E Spinning Disk confocal microscope *en montage* using the focus surface interpolation tool in Nikon Nis-Elements AR 5.21.03. This image served as a map to direct imaging of individual cells and also to mark their coordinate location in the flattened retina. Z-stacked images were taken of individual astrocytes at 60X magnification through the entirety of each cell’s volume at a step size of 0.3 μm. Images for each channel were taken serially at each Z-step.

### Computation

Morphology was quantified for astrocytes which met the criteria of being V5-positive, GFAP-positive cells with cell bodies residing in the Nerve Fiber Layer (NFL) or Ganglion Cell Layer (GCL). Cells were not included in analysis if they were in contact with any other V5-labeled astrocyte or could not have their outline determined accurately. Using this exclusion criteria, just over 1,000 cells were analyzed.

Cartesian coordinates for cells on flat-mounted retinae were mapped to geographic coordinates in native retinal space using the open-source program RETISTRUCT (Sterratt et al., 2013). It and its dependencies were installed onto a machine running Windows 10 using a Microsoft Image of the CRAN from 02/11/2019, R version 3.4.2, and Rtools35. Using the fluorescein angiograms alongside the 20X IB4 montages, retinal orientation was determined. This information along with cartesian coordinates of each cell were input into RETISTRUCT. The φ0 angle used during reconstruction was 22 degrees. Picturing the retina as a hemisphere, the rim (far periphery) is located at 0 degrees latitude and the ONH is located at −90 degrees. Longitude is taken such that zero degrees is temporal for left eyes and nasal for right eyes. 90 degrees longitude is always dorsal. The raw data outputting latitude and longitudes of each cell was read into a custom Python program which translated coordinates for all right eyes into a common left-eye coordinate space.

Cell outlines were generated using a semi-automated, custom ImageJ macro. All functions referenced are available in Fiji ImageJ version 2.3.0 on Windows operating systems. This program flattened a V5 labeled astrocyte Z-stack using the standard deviation Z-stack function. This flattened image was then automatically thresholded and binarized. Outlines of cells were acquired using the Particle Analyzer Tool looking for particles with size bounds of 3 μm^2^ to infinity. ROIs were then generated, and a user scanned through them to find ROIs that either contributed to the cell’s outline or its holes. These ROIs were then subjected to the XOR operation to get the final complex cell outline. Using this macro to determine outlines takes about 1 min or less per cell. After the complex cell outline was generated, its convex hull was also generated. Each of these outlines were subjected to shape analysis using the following parameters in the ‘Set Measurements’ window: [area, center of mass, shape descriptors, area fraction, centroid, perimeter, fit ellipse, and Feret’s diameter]. The GFAP channel was processed in a similar way, except that binarization was achieved using the Phansalkar Auto Local Threshold function with a radius of 15. This automatic thresholding was sufficient for all images. Throughout this paper, projection area is used to refer to the area of V5-positive labeling in a Z-projected astrocyte image. Cell outlines and binarized GFAP images were used to determine percent GFAP-positive projection area.

Distance from each cell’s convex hull centroid to the closest blood vessel was determined using a custom Python script. This script read in the convex hull information from the ImageJ macro results and displayed a standard deviation Z-projected image to a user showing the V5 and IB4 channels. A circle was overlaid on the image centered at the centroid, and an adjustable slider changed the radius of this circle. A user adjusted the slider until the edge of the circle touched the closest vessel and then this value was recorded. Similarly, the diameters of unique vessels contacted were determined using another Python script with the same V5/IB4 images. In this program a user noted the boundary points of each unique vessel and their diameters were displayed and recorded. Unique vessels are defined after branching events (where each branch results in one continuing vessel and one new one). Only the number of unique contacts was quantified- the total number of contacts which includes repeated contacts to the same vessel was not.

Visualization of all data was accomplished using the Python library Matplotlib and GraphPad Prism. BioRender was used to generate the image of the Micron IV and retinal vasculature in Supplementary Figure S2. All other figures were crafted in Adobe Illustrator and Python. UMAP (Uniform Manifold Approximation Projection) was run using the Python library UMAP-Learn with n_neighbors = 15. Running UMAP with a variety of n_neighbors values yielded similar embedding shapes. Thus, the default value of 15 was chosen. The 20 astrocyte morphology parameters that were subjected to the UMAP algorithm include: Full-Cell outline (FO) area, FO perimeter, FO major axis, FO minor axis, FO circularity, FO Feret diameter, FO roundness, FO solidity, area of the convex hull (CVH) of the FO, CVH perimeter, CVH Feret diameter, latitude (radians), longitude (radians), percent of FO projection area that is GFAP-positive, distance from the CVH centroid to the nearest blood vessel, the distance from the CVH centroid to the FO center of mass, mean vessel diameter for unique vessels contacted, minimum vessel diameter for unique vessels contacted, maximum vessel diameter for unique vessels contacted, and the number of unique vessels contacted. The UMAP embedding was clustered by density using Hierarchical Density-based Spatial Clustering of Applications with Noise (HDBSCAN). HDBSCAN parameters used were min_samples = 3 and min_cluster_size = 10. The embedding was also clustered according to each individual UMAP input parameter.

Data and code not explicitly shown in this manuscript are available upon request to the corresponding author.

## Results

### S100β, V5, and dye filling reveals that most astrocytes do not exhibit stellate morphology in the retina

**Figure 1:**
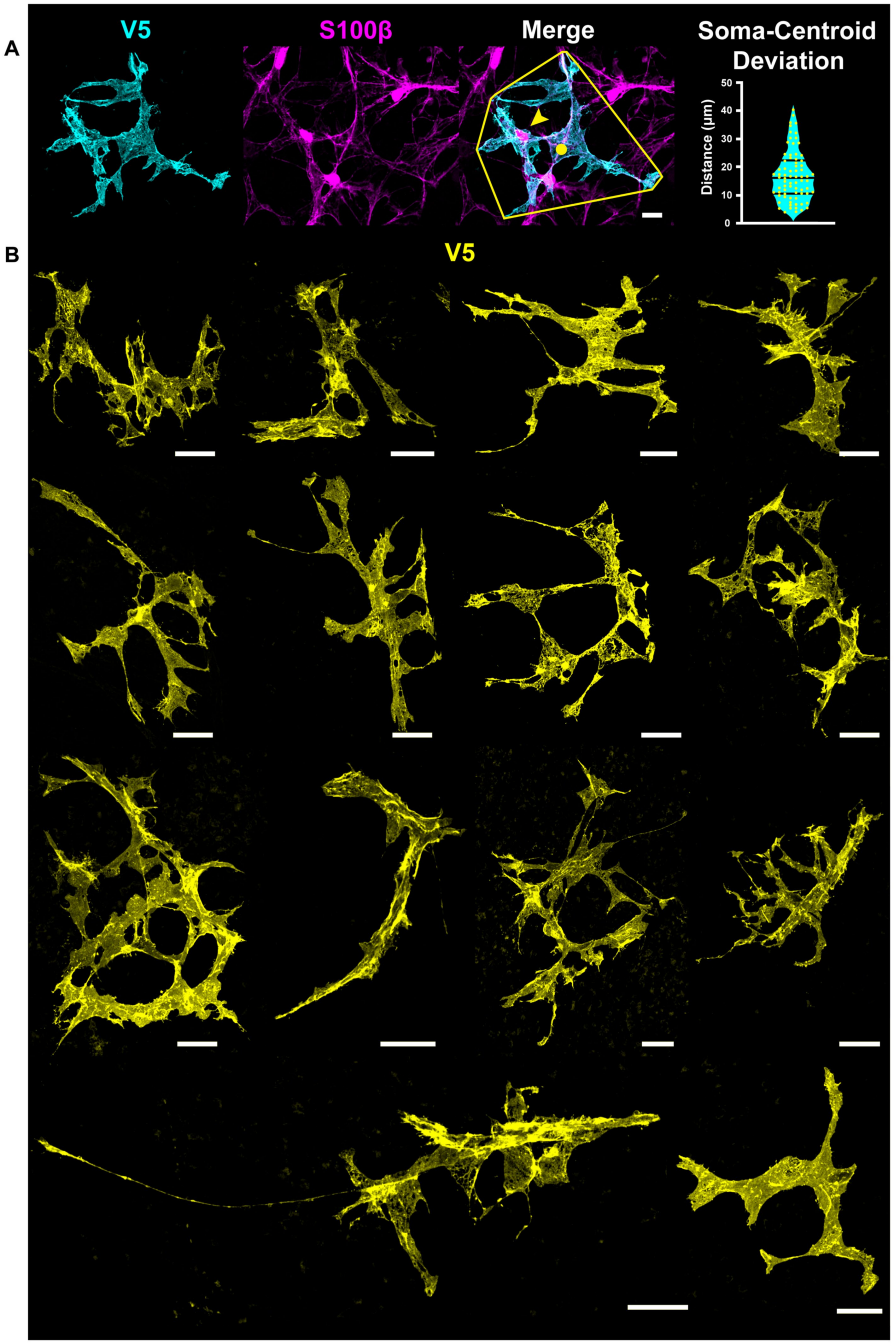
Not a Star – S100β, V5, and dye filling reveals that most astrocytes do not exhibit stellate morphology in the retina. Stellate or star-shaped morphology refers to a cell with a centrally located soma with processes emanating from it in all directions. This is not an appropriate description for the majority of retinal astrocytes. **(A)** Astrocyte soma location (S100β, yellow arrow) is often displaced relative to the centroid of its convex hull (yellow circle). The violin plot shows the distance between the soma and convex hull centroid. The average separation is 17.1 μm (STDEV = 9.1 μm, SEM = 1.1 μm. *N* = 74 cells). Scale bar indicates 15 μm. **(B)** Examples of astrocytes with non-stellate morphology in the retina using G-MORF mice. Each image is of a single astrocyte, confirmed by S100β. Scale bar indicates 25 μm.

Cells with stellate morphology have a central cell body which radiates processes uniformly in all directions, much like a star. We overcome limitations of current astrocyte visualization techniques using the G-MORF mouse line and find that this description does not accurately describe most retinal astrocytes (Supplementary Figures S1–S3). Even cells which appear stellate from V5 labeling alone commonly have their cell body in an unexpected location toward the periphery of the cell mass (Figure 1A). S100β prominently labels the soma, which is often far from the centroid of the cell’s V5 convex hull (Figure 1A, ${\text{distance}}_{\text{centroid}-\text{soma}}=17.1\pm 9.1\;\mu m,\text{mean}\pm \text{stdev}$). Example cells from G-MORF mice exhibiting non-stellate morphology are shown in Figure 1B. Immunolabeling for NLRP3 inflammasome and microglial ionized calcium-binding adapter molecule-1 (IBA-1) did not show signs of immune activation from smFP expression which could affect astrocyte morphology in G-MORF mice (data not shown).

### GFAP and S100β underestimate astrocyte complexity

**Figure 2:**
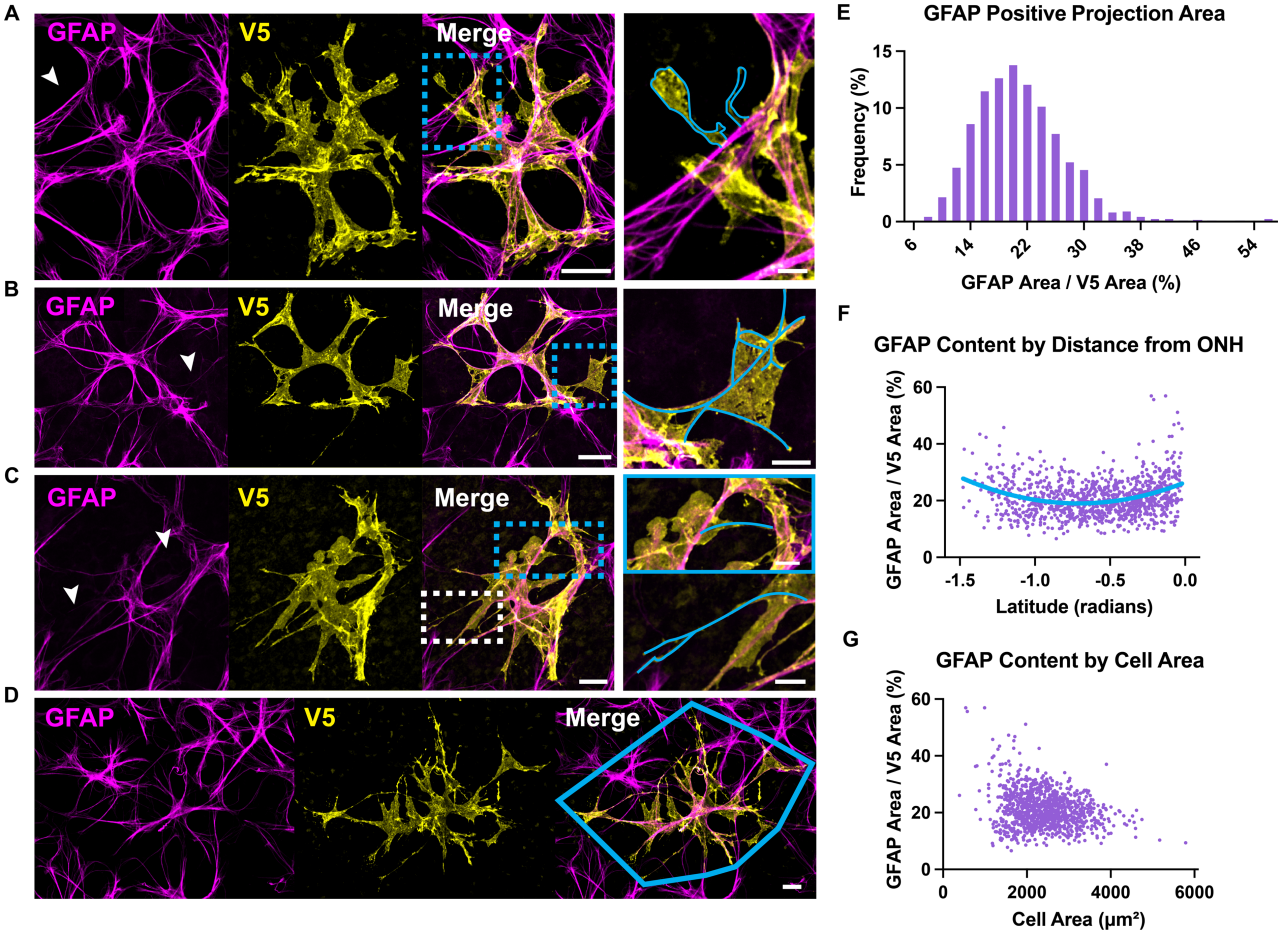
GFAP underestimates astrocyte complexity. **(A)** Some astrocyte processes are GFAP-negative. An example process devoid of GFAP is outlined in cyan. Full-size image scale is 25 μm and zoomed inset is 5 μm. **(B)** GFAP fails to predict membranous morphology. Inset shows trace of GFAP skeleton in cyan. Full-size image scale is 25 μm and zoomed inset is 10 μm. **(C)** Faint GFAP-positive staining in processes is easily overlooked. Inset shows GFAP skeletons traced in cyan for selected processes that are obscured in the large figure. Arrows indicate processes which appear to terminate or are completely absent in the GFAP channel until image enhancement. Full-size image scale is 15 μm and zoomed inset is 7 μm. **(D)** Retinal astrocyte domains often overlap extensively, meaning it is difficult to assign GFAP signal to a single cell. Cyan outline indicates convex hull of V5-labeled cell. Scale bar indicates 15 μm. **(E)** GFAP accounts for 21.1 ± 0.2% of overall cell area meaning its signal misses close to 80% of cell morphology. GFAP also makes up a larger percent of projection area at the ONH and peripheral retina. As cell area increases, GFAP accounts for less of that area. **(F)** GFAP content varies with distance from the optic nerve head. At the ONH and far periphery, GFAP makes up a greater percentage of cell area. **(G)** GFAP content varies with cell size. Larger cells tend to have a smaller fraction of GFAP area to V5 area. Scale bar indicates 25 μm, *N* = 1,045 cells.

**Figure 3:**
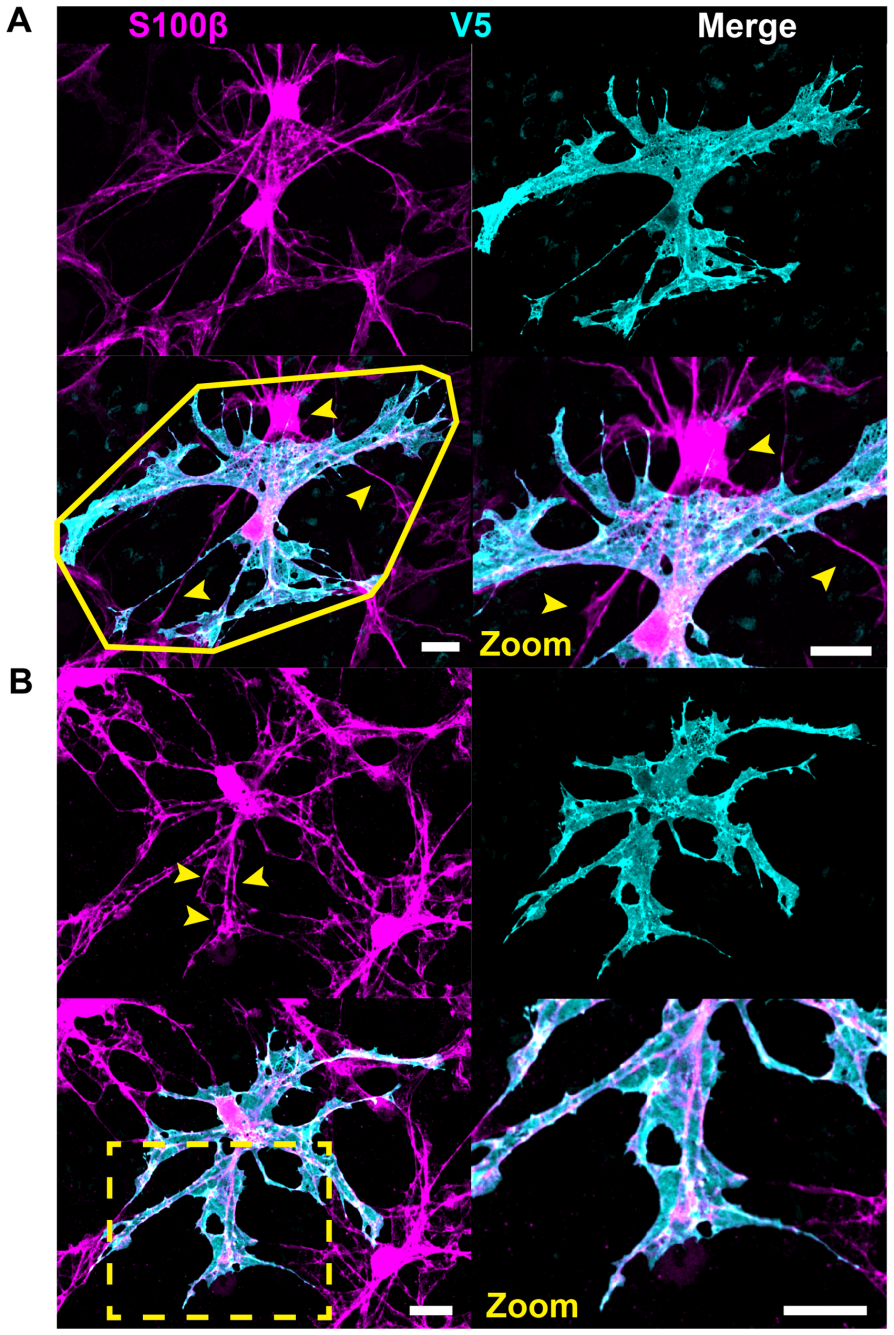
S100β cannot resolve individual cell morphology. **(A)** S100β cannot reveal individual cell boundaries due to the extensive spatial domain overlap. Yellow polygon indicates convex hull. Arrows indicate regions of V5-negative cell infiltration. **(B)** S100β does not uniformly label V5-positive area. Rather it is prominently associated with the cytoskeleton and weakly with the membrane. It also strongly labels the cell body. Yellow arrows and zoomed inset reveal cytoskeletal labeling and lack of full membranous labeling. Scale is 15 μm.

Astrocyte morphology revealed using immunolabeling against GFAP and S100β underestimates actual complexity as shown by V5. Some V5-labeled processes in G-MORF mice are GFAP-negative (Figure 2A), and many membrane protrusions are relatively planar rather than fibril-like (Figure 2B). Low levels of GFAP also obscure finer processes (Figure 2C). Retinal astrocyte spatial domains often overlap extensively (Figures 2D, 3A), indicating that pan-astrocyte markers alone cannot distinguish individual cells. GFAP signal represents a relatively small proportion of a cell’s projection area (21.1 ± 0.2%, Figure 2E), which depends on both cell size and retinal location (Figures 2F–G). Its content also varies with eccentricity, accounting for a larger percentage of cell projection area at the nerve head and in the far periphery and a smaller fraction as cell size increases. S100β similarly cannot reveal individual cell morphology. It does not uniformly label V5-positive area and is more prominently associated with the cytoskeleton than with cell membrane or cytosol (Figure 3B).

### V5 labeling reveals structural motifs

**Figure 4:**
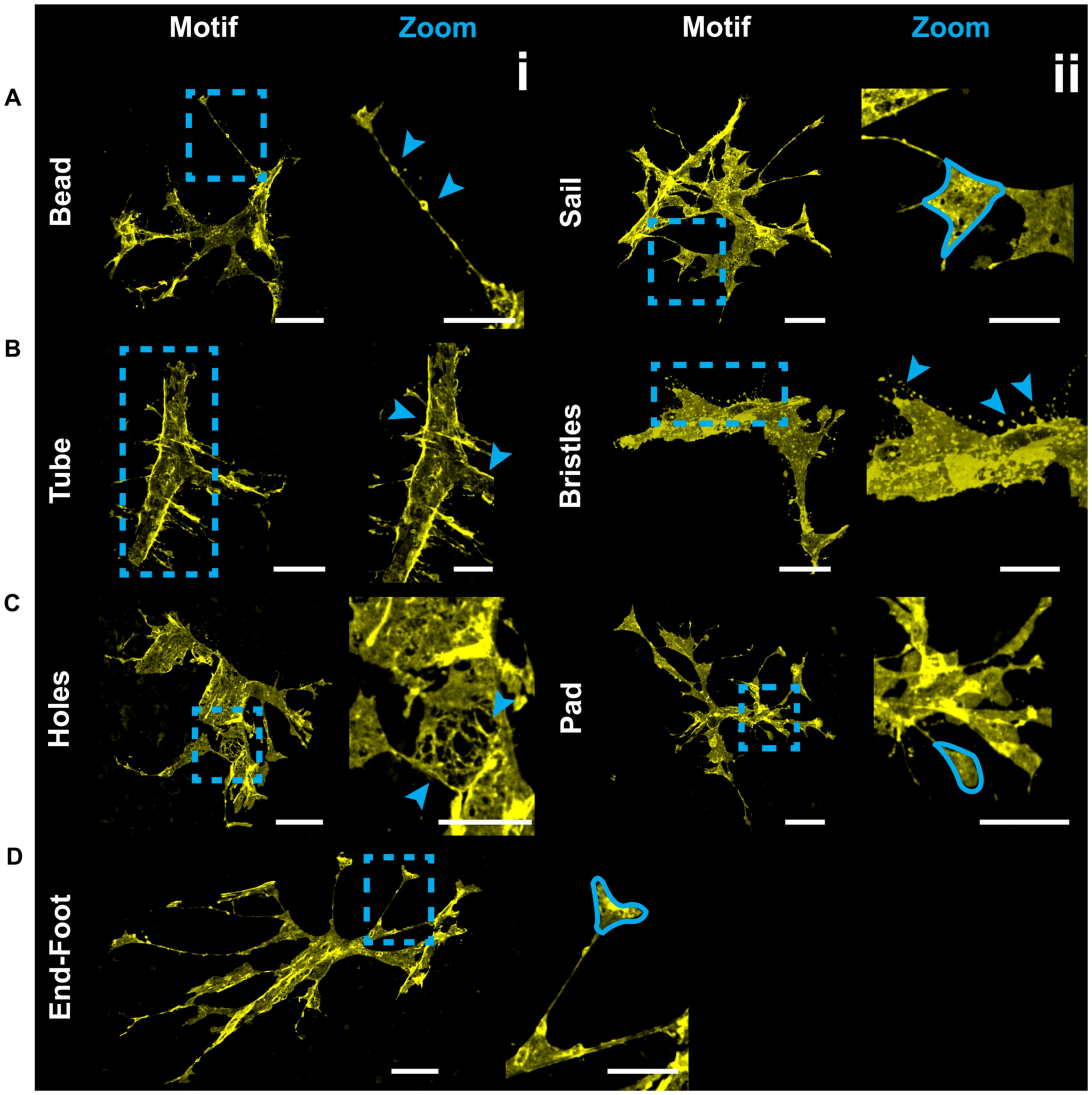
V5 labeling reveals structural motifs. Seven structural motifs are shown, and each astrocyte exhibits at least one of them. Cyan dashed boxes in eachmotif column indicate the region expanded in the zoom column. Arrows and tracings indicate each motif in the zoomed region. Row **(A)** highlightsboth the bead motif (i) as well as the sail motif (ii). Row **(B)** highlights both the tube (i) and bristle (ii) motifs. Row **(C)** highlights both the holes (i) and pad (ii) motifs. Row **(D)** highlights the end-foot motif (i). Large image scale 25 µm, inset zoom scale 15 µm.

**Figure 5:**
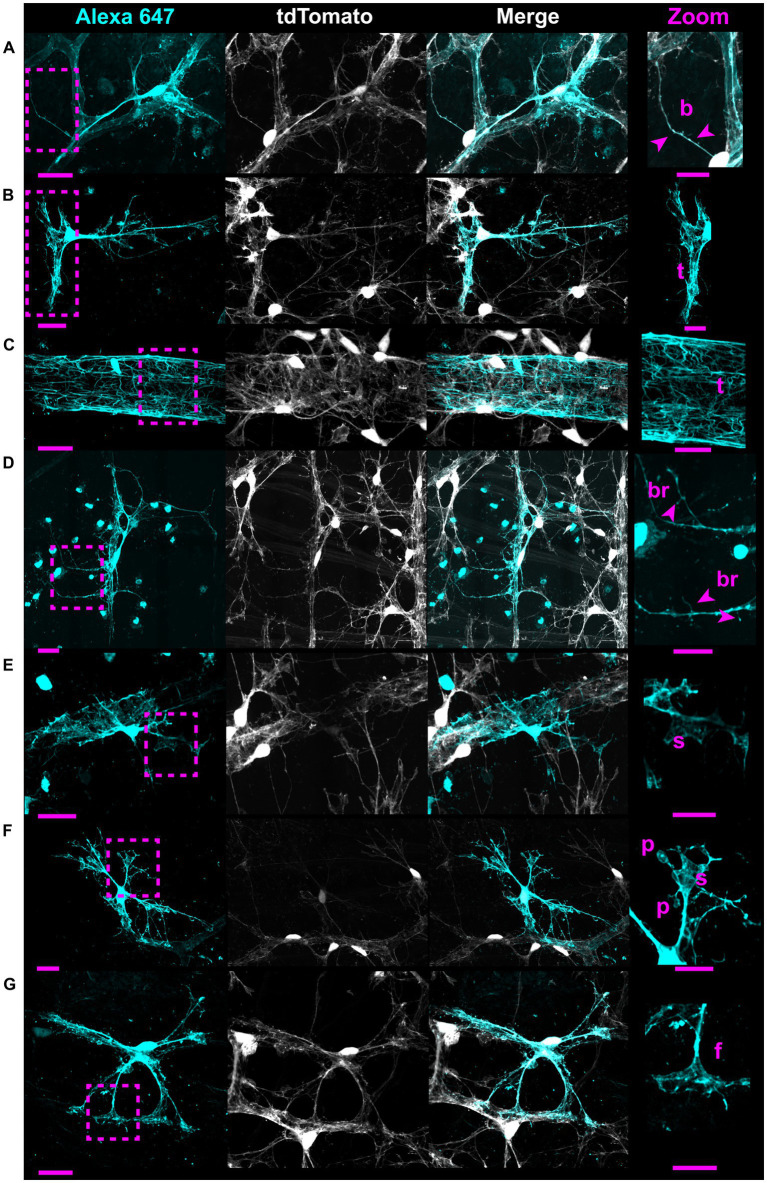
Whole-cell dye filling reveals motifs and non-stellate morphology. Each row shows an astrocyte from a Cre 77.6 / tdTomato mouse patched in a whole-cell configuration and filled with Alexa 647. As it is a gap junction permeable dye, labeling of adjacent astrocyte soma and ghost-labeling of blood vessels is sometimes visible. These fillings reveal the same motifs as astrocytes from G-MORF mice. From **(A–G)**, rows highlight beads “b,” a small tube motif “t,” a large tube motif “t” on an astrocyte making an enveloping connection, bristles “br,” sails “s,” pads “p,” and end feet “f.” Astrocytes in rows **(A–E)** have morphology that does not fit the traditional stellate description. Scale bar of the full-size images is 25 μm and for the zoomed images is 15 μm.

V5 labeling reveals recurring astrocyte structural motifs, which we now define. Beads are small, ellipsoid and triangular inflations of cell membrane along thin projections (Figure 4Ai). Sails are polygonal sheets of membrane that often have thin projections emanating from their vertices (Figure 4Aii). These can either be small motifs or encompass the majority of the cell volume. Tubes form as an entire cell or its processes envelop a blood vessel (Figure 4Bi). Bristles are short, fine projections on the order of 5 μm in length (Figure 4Bii). The smallest bristles often terminate in a roughly spherical shape and are between 0.7 and 2.5 μm in length. Pads and end-feet are morphologically similar but are distinguished based on which structures they contact. We define pads as rounded, flat projection endings which do *not* contact blood vessels, whereas end-feet terminate on blood vessels but do not envelop those vessels completely (Figures 4Cii,Di). We also observed that holes commonly form within large sheets of membrane (Figure 4Ci). These motifs align with morphology observed in whole-cell dye filling (Figure 5).

### Structural motifs predict underlying vascular and neuronal architecture

**Figure 6:**
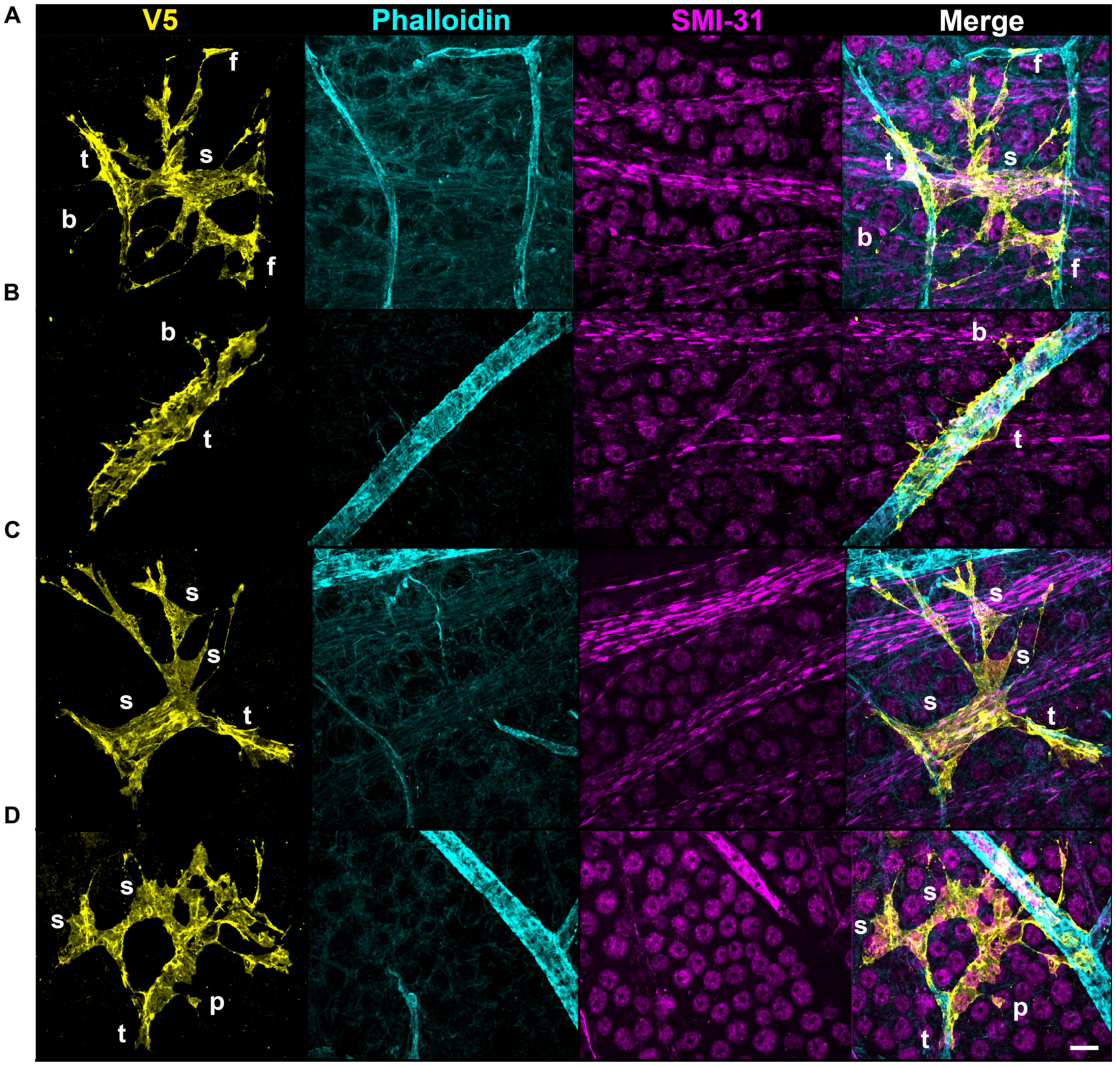
Structural motifs predict underlying vascular and neuronal architecture. Select motifs from Figure 4 are highlighted here with underlying vascular and neuronal structures. **(A)** In this astrocyte a sail motif terminates on the left at a blood vessel where it wraps around to form a tube motif. On the right, along with other processes, the sail terminates as end-feet on blood vessels. Additionally, a thin process encircles an RGC soma which has beads. **(B)** The major motif in this astrocyte is the tube. A small process also emanates from the tube which terminates as a bead on an RGC axon. **(C)** This astrocyte has prominent sails. They cover axon bundles and the apical surface of RGC soma. **(D)** In this astrocyte, sails cover RGC soma. The membrane shape of the sails predominantly covers these soma and not the negative space between cells. There is also a pad that contacts an RGC soma near the axon hillock region. Scale is 15 μm. Motifs are identified using the first letter in their name using white letters.

**Figure 7:**
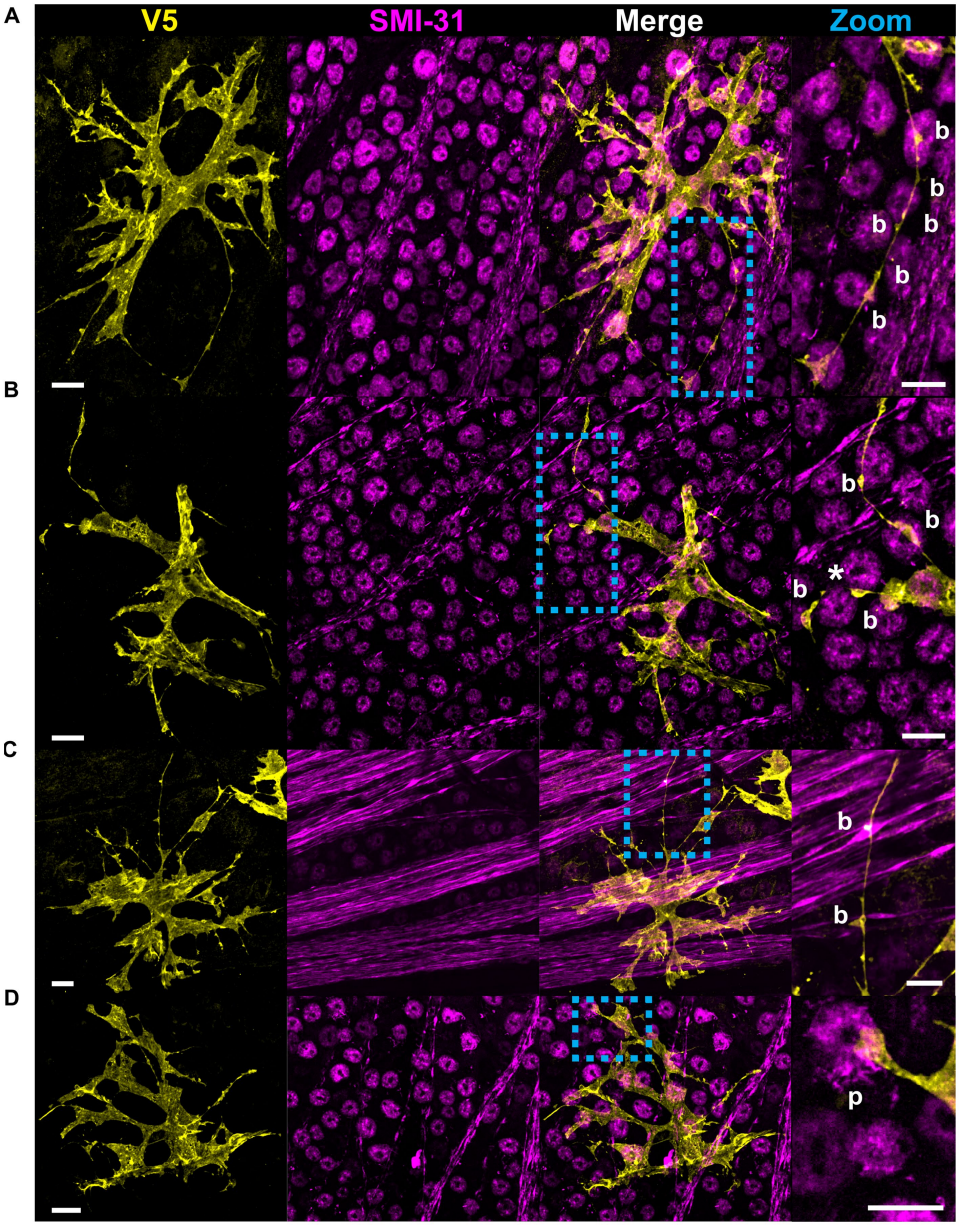
Beads and pads contact RGC axons and cell bodies. **(A)** In this example, beads (indicated with “b”) contact every soma surface that the process traverses. **(B)** Beaded processes can encircle the lateral surface of RGC soma, indicated with an asterisk. **(C)** Beads also contact RGC axons, often coinciding with varicosities in the axons themselves. **(D)** Pads (indicated with “p”) form contacts with RGC soma. These contacts are found at the apical surface of the RGC soma only. Scale for full images is 15 μm and for zoom insets is 10 μm.

**Figure 8:**
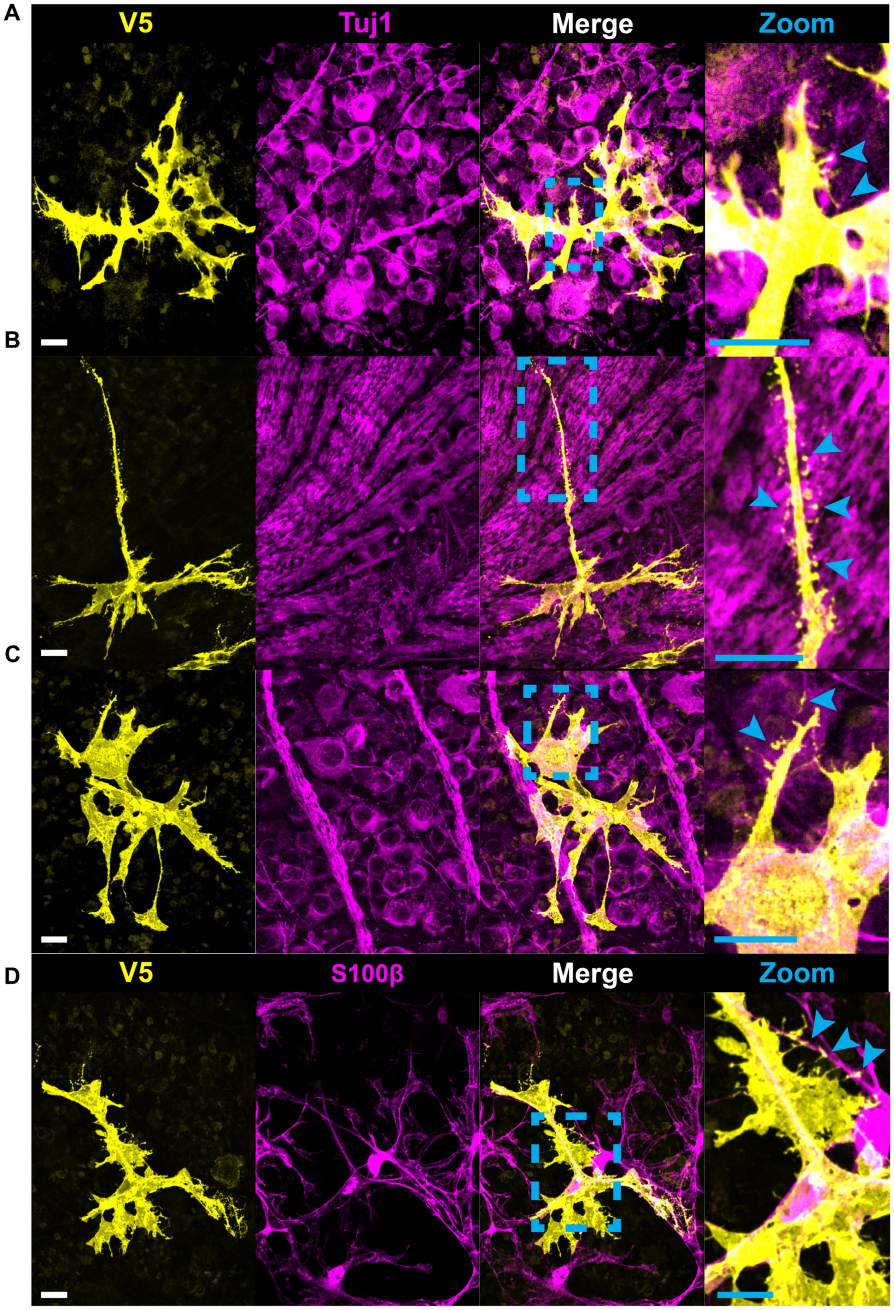
Astrocytes make fine connections to single axons, RGC soma, and other astrocytes through the bristle motif. **(A)** Astrocytes make connections to single axons through bristles. These occur at segments of axon both with and without varicosities (cyan arrows). **(B)** Astrocytes close to the optic nerve head extend bristles which make contacts to axons across distinct fascicles. This type of interaction is also common on astrocytes which envelop large blood vessels and make contacts to nearby axon bundles. **(C)** Astrocytes contact RGC soma through bristles. These bristles commonly trace individual axons (cyan arrows). **(D)** Astrocytes also use bristle motifs to contact nearby astrocyte processes. Scale bars indicates 15 μm.

Structural motifs are predictive of the underlying architecture in the Nerve Fiber Layer (NFL) and Ganglion Cell Layer (GCL). In Table 1 we quantify this predictive capacity by determining the probability of observing an underlying structure given the independent observation of an astrocyte motif, p(structure|motif). Sails are predictive of both RGC soma (41%) as well as bundles of RGC axons (65%) and weakly predict neighboring astrocytes (6%) (Figures 6A,C,D). As membrane sheets, they cover large regions of axon bundles but do not envelop them completely. They may also cover one or more RGC soma. Tubes are predictive of underlying blood vessels (100%), only occurring when one is present at its interior (Figure 6B). End-feet are morphologically similar to pads, with the distinction being their association with blood vessels and not RGC soma. As they are defined based on their vascular interaction, p(vessel|endfoot) = 100%. Their morphology can be roughly flat and circular like pads or can be more curved to complement the curvature of the vessel itself (Figures 4D, 6A). Similarly, they may terminate at a blood vessel as a larger mass of membrane that does not have a very distinct shape (Figure 6D). Beads are most predictive of axons (76%) but also contact RGC soma (22%). When contacting soma, we observed bead placement on the apical surface close to the hillock region as well as intercalating between the lateral surfaces of adjacent RGC soma (Figures 6A, 7A,B). When contacting axons, we observed beads often contacting varicosities on those axons (Figure 7C). Pads similarly contact RGC axons (43%), soma (43%), and other astrocytes (20%). Unlike beads, pads were only observed to contact the apical surface of RGC soma (Figure 7D). Bristles contact individual RGC axons (41%) and soma (34%) as well as neighboring astrocytes (20%) (Figure 8). They contact segments of axon with and without varicosities (Figure 8A) and often trace individual axons for the entire length of the motif (Figures 8A,C). Bristled processes can additionally span multiple axon bundles at the optic nerve head, making fine connections to axons in each fascicle (Figure 8B).

**Table 1:** Quantifying predictive power of non-vascular-associated motifs.

	Axon	RGC Soma	Astrocyte	Error
Sail	65%	41%	6%	7%
Bead	76%	22%	3%	5%
Pad	43%	43%	20%	13%
Bristles	41%	34%	20%	15%

Each table entry represents the probability of observing a neuronal or astrocytic structure given a motif is present p(structure|motif). The error column represents the probability that an astrocytic or neuronal structure is not observed where a motif is present. These motifs are most predictive of neuronal structures, especially axons. Pads and bristles are also commonly associated with neighboring astrocytes. The vascular end-foot and tube motifs are defined based on their vascular contacts so p(vessel|tube)=100% and p(vessel|end−foot)=100%. The number of cells quantified for this table that exhibited sails, beads, pads, or bristles was 69, 37, 45, and 33. The total number of motifs quantified for sails, beads, pads, and bristles was 231, 203, 101, and 388.

### All astrocytes directly contact vascular and neuronal structures

**Figure 9:**
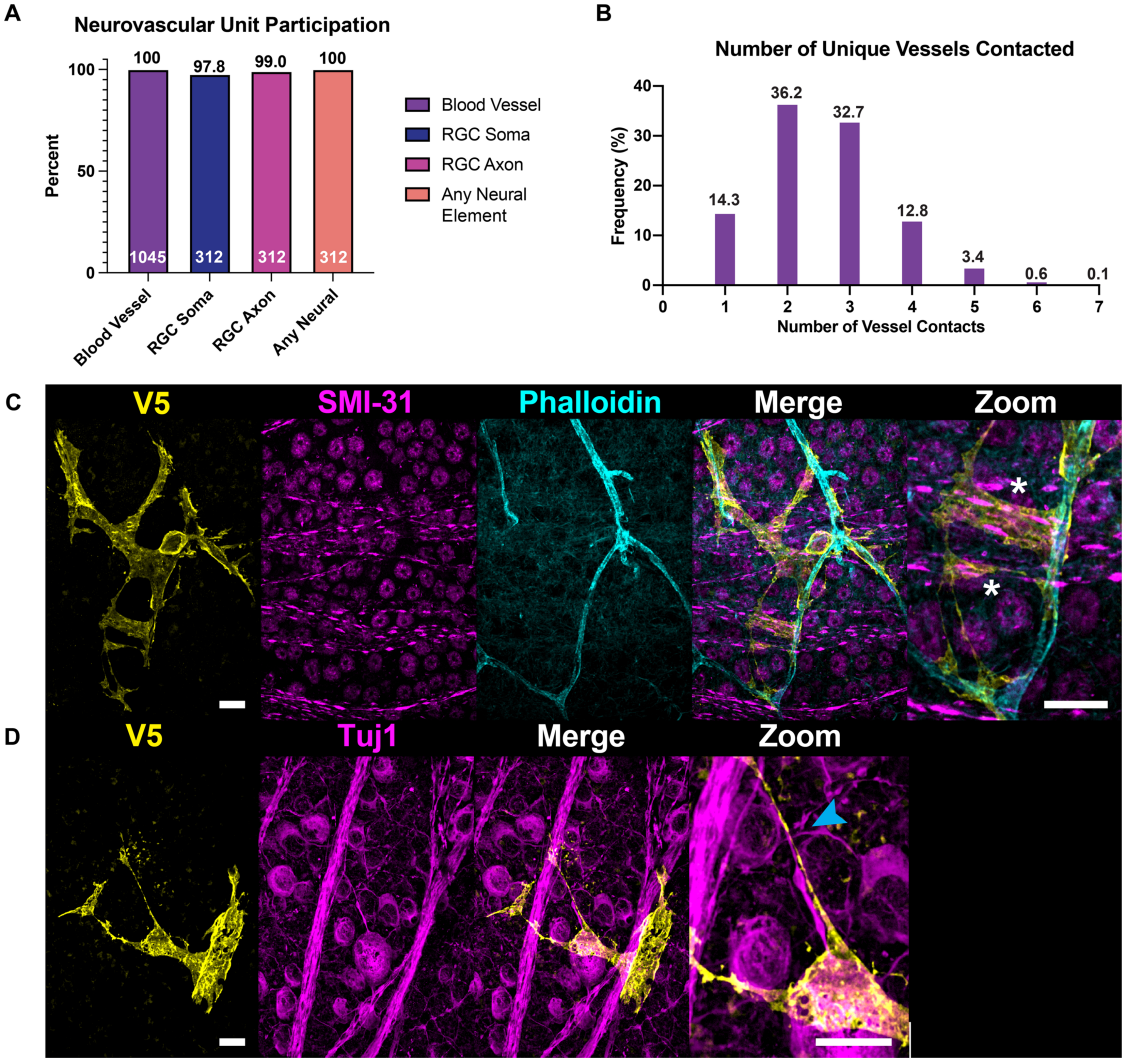
All astrocytes directly contact vascular and neuronal structures. **(A)** 100% of astrocytes contact at least one blood vessel (*N* = 1,045 cells). 100% of astrocytes also contact some neuronal element (SMI-31 or Tuj1-positive structure, *N* = 312 cells), whether that is an axon or an RGC soma. 97.8% of astrocytes contact RGC soma and 99.0% contact axons. The cells that did not contact RGC soma were found in regions of high-density axon bundles where RGC soma were not found in image stacks. Additionally, some cells which completely enveloped blood vessels did not contact RGC soma. Astrocytes which did not contact axons were found in regions of the retina which had no axons labeled by SMI-31 or Tuj1. **(B)** Frequency distribution for the number of unique vessels contacted by a single astrocyte. Average number of unique contacts was 2.57 ± 0.03 (*N* = 1,045). **(C)** Astrocyte processes often follow axon bundles in their ultimate path to contact blood vessels, indicated by an asterisk. **(D)** Shows an astrocyte process which traces an RGC axon as it fasciculates into a nerve fiber bundle. Sale bars indicate 15 μm.

We used co-labeling of V5 and IB4 to determine whether morphological differences would be found between astrocytes exhibiting motifs interacting with vasculature and those which did not. To our surprise, every astrocyte contacted at least one blood vessel, with most cells contacting multiple unique vessels (Figures 9A,B). On average, astrocytes contacted 2.57 unique vessels (Figure 9B). We similarly found that every astrocyte contacted some neuronal element in the GCL or NFL, be it an axon or soma (Figure 9A). Additionally, individual astrocyte processes were not limited to making mutually exclusive contacts to blood vessels or neuronal elements. Rather, many processes contacted both (Figure 9C). In these instances, both axons and blood vessels appeared to act as tracks guiding processes to one another. We observe a similar interaction between astrocyte processes and axons undergoing fasciculation (Figure 9D).

### Astrocytes interact with the vasculature in three ways

**Figure 10:**
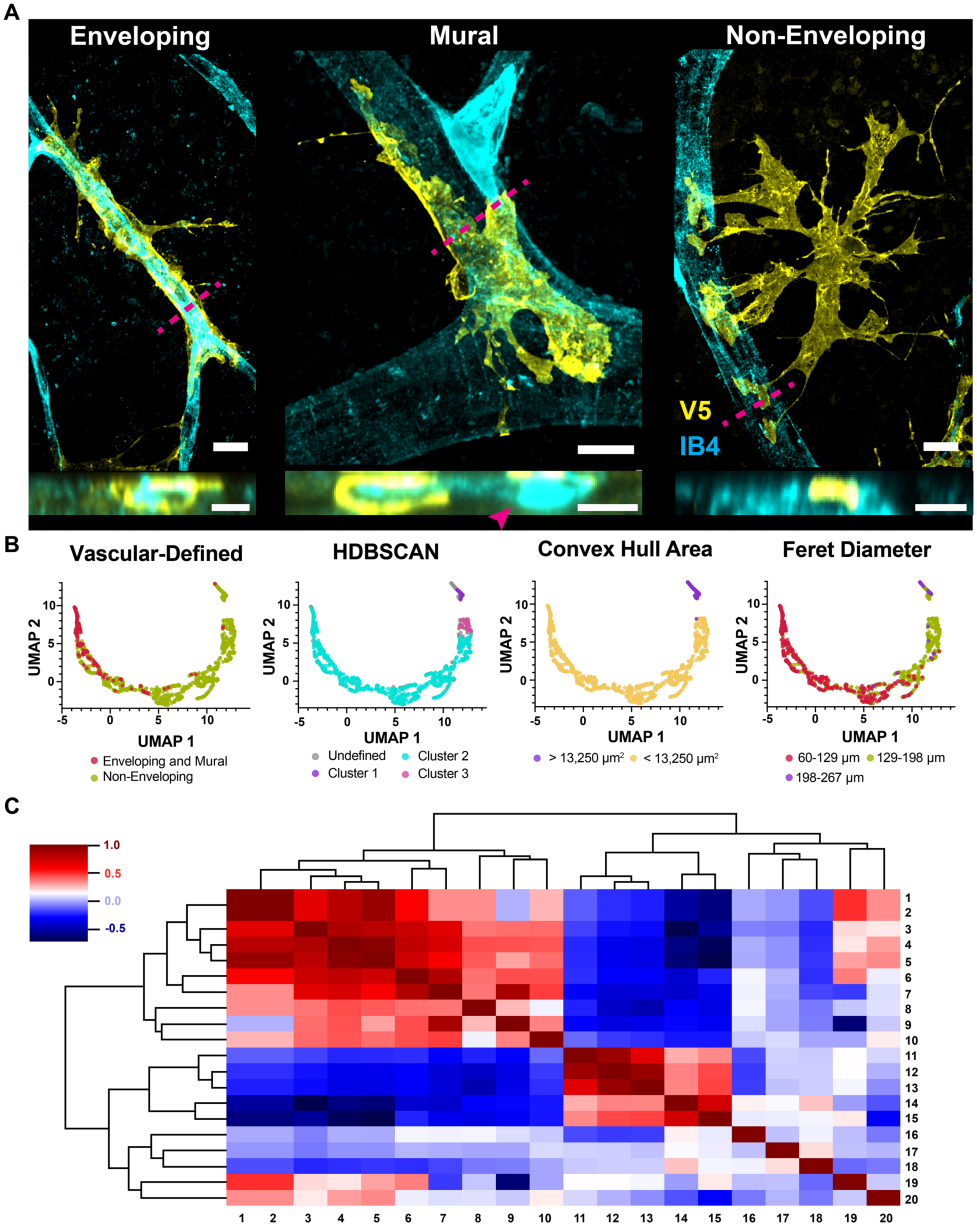
Astrocytes interact with the vasculature through three mutually exclusive types of contacts. **(A)** Astrocytes interact with the vasculature in three ways. Shown are the enveloping, mural and non-enveloping contacts. Top view is *en face* and bottom is a cross section reconstruction from confocal stacks. Magenta dashed line indicates where the cross-section plane was taken. Scale bar indicates 15 μm. **(B)** UMAP embedding plots were clustered by the vascular connection type, density (HDBSCAN), convex hull area, and Feret diameter. **(C)** Correlation dendrogram showing relationships between all parameters used in the UMAP. The plot is organized such that similar rows and columns are arranged close together. Each parameter is aliased by a number. These parameters are: 1. FO Feret diameter, 2. CVH Feret diameter, 3. FO perimeter, 4. CVH area, 5. CVH perimeter, 6. FO area, 7. FO minor axis length, 8. number of vessel contacts, 9. FO roundness, 10. distance to closest vessel, 11. max vessel diameter contacted, 12. mean vessel diameter contacted, 13. min vessel diameter contacted, 14. FO circularity, 15. FO solidity, 16. latitude, 17. longitude, 18. FO percent GFAP coverage, 19. FO major axis length, and 20. the distance between the FO center of mass and the CVH centroid. Full-outline of the astrocyte projection is abbreviated FO and its convex hull is abbreviated CVH.

**Figure 11:**
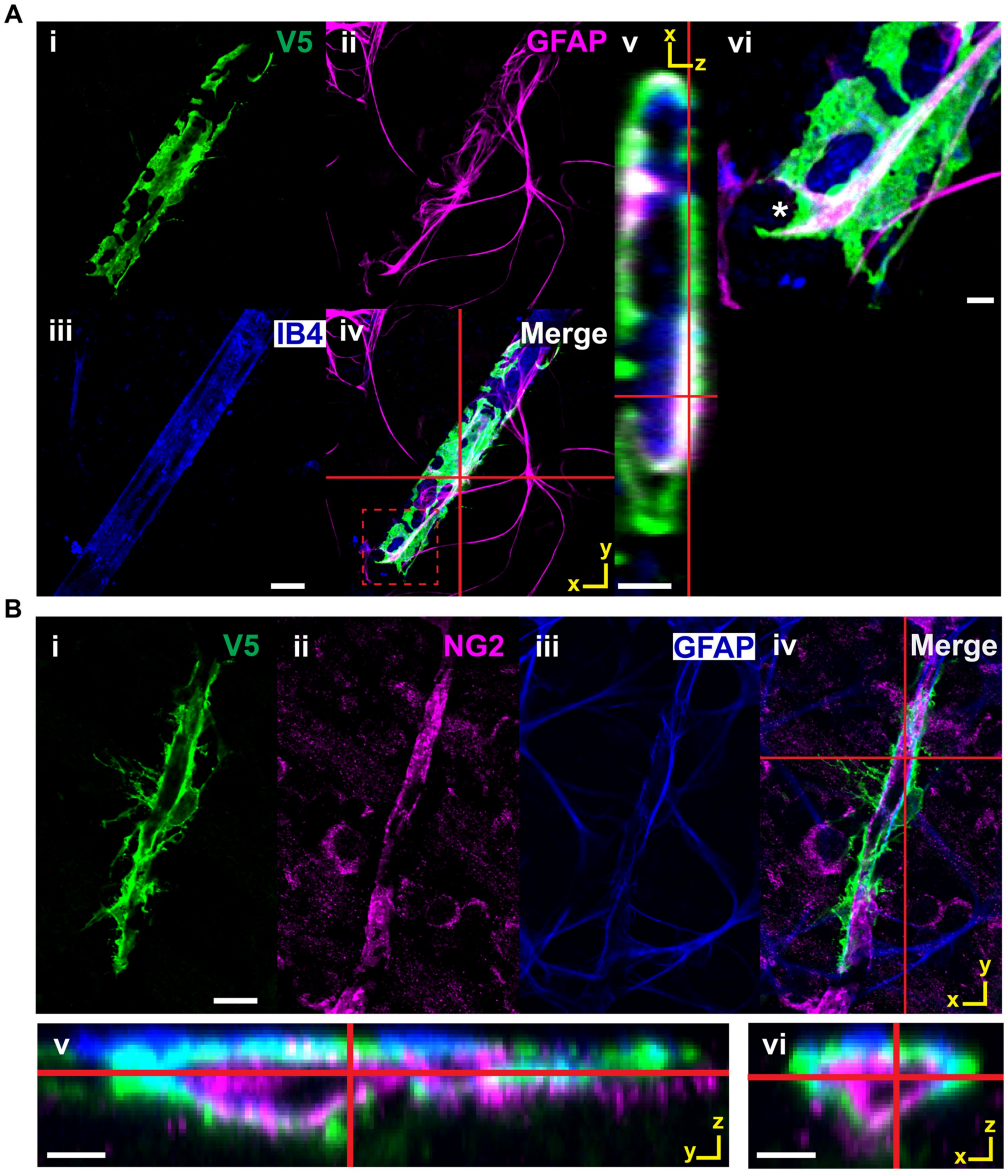
V5-positive cells making enveloping contacts are astrocytes, not pericytes. **(A)** V5-positive cells making enveloping contacts are GFAP-positive. i–iv show such an astrocyte labeled with V5 along with GFAP and IB4. Red cross hairs indicate where the XZ orthogonal plane is taken for inset v. Red dashed square indicates the region shown in close-up in vi. GFAP clearly defines the structure of the membrane segment defined by the V5 label. Scale bar for i-iv is 15 μm and for v-vi is 3 μm. **(B)** Cells making enveloping connections are NG2-negative and GFAP-positive. Panels i-iv show an astrocyte (V5, green) which is wrapping around a pericyte (NG2, magenta). Red cross hairs indicate the point at which the XZ and YZ planes were taken to construct the orthogonal views in v-vi. Scale bar for i–iv is 15 μm and for v-vi is 3 μm.

All astrocytes interact with the vasculature through one of three mutually exclusive connection types (Figure 10A). Cells with enveloping connections appear as tubes which completely enwrap both major vessels as well as capillaries, with either few or no processes extended. They represent 8% of analyzed cells. To ensure they were not vascular pericytes, we co-labeled retinae for chondroitin sulfate proteoglycan (NG2), V5 and GFAP. V5-positive cells making enveloping connections are NG2-negative and GFAP-positive (Figure 11). The mural connection type is similar, with these cells only partially enveloping the vasculature. They represent 7% of analyzed cells and are commonly found at the junction of diverging vessels. Enveloping and mural contacts are made with the majority of the cell’s mass. Finally, 85% of cells make non-enveloping contacts. These include end-feet and small tube motifs.

These vascular connections describe overall cell shape and explain a significant portion of morphological heterogeneity. We quantified morphological features for over one thousand astrocytes and reduced that feature space to two dimensions using UMAP. Classes of vascular interactions, when applied to color the embedding, are predictive of plot structure with the caveat that distinguishing between cells making mural and enveloping connections did not provide additional prediction power (Figure 10B). Density-based clustering of the embedding revealed three populations of cells which do not directly correspond to vascular-defined classes (Figure 10B). We also colored the embedding according to each feature used as a UMAP input parameter (Figure 10B) which revealed that plot structure is explained in part by cell size and polarization (Feret diameter and minor axes). Cluster 1 contains cells with a large convex hull area and long Feret diameter, while drivers of Cluster 2 and 3 were not apparent. A dendrogram showing all correlations between UMAP input parameters is shown in Figure 10C.

### Astrocytes are compressed near the ONH and far periphery

**Figure 12:**
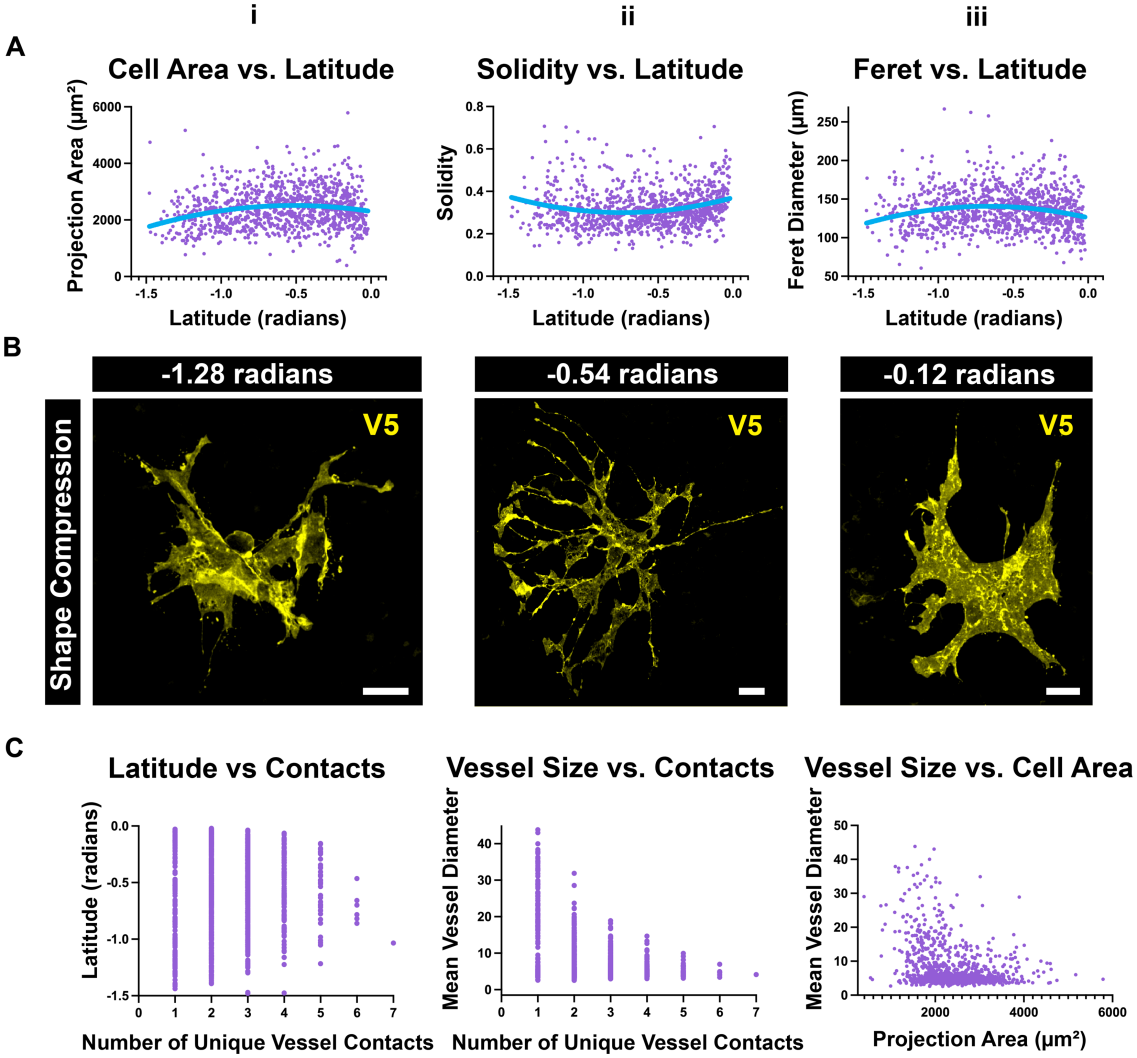
Peripheral and ONH astrocytes exhibit a compressed morphology **(A)** i. Population-wide projection area is maximal in the mid-retina. ii. A complementary relationship exists with solidity. iii. Population-wide Feret diameter is maximal in the mid-retina. **(B)** Select astrocytes at various latitudes from the ONH (in radians). These images demonstrate compression in astrocyte shape at the peripheral retina and ONH compared to the mid-retina. Scale 15 μm. **(C)** i. The mid-retina is the region where astrocytes are most likely to contact many vessels. ii. Astrocytes that make many unique contacts are more likely to contact smaller vessels than large ones. iii. Cells that contact larger vessels are more likely to be smaller cells.

Using the data collected for UMAP, we separately investigated whether astrocyte morphology varied with retinal location. We plotted individual morphological parameters against geographic coordinates for each cell in native retinal space. Latitude provides information regarding distance from the ONH whereas longitude provides information about retinal region (nasal, temporal, dorsal, ventral). Longitude was not predictive of any parameter that was quantified; however, many varied with latitude. When plotted against latitude, cell area and Feret diameters exhibit a slight negative curvature while solidity shows a slight positive curvature (Figure 12A). This indicates that the populations of astrocytes near the ONH and far periphery are compressed in shape relative to cells in the mid-retina (Figures 12A,B). Additionally, astrocytes in the mid-retina are more likely to contact larger numbers of unique blood vessels than their counterparts at the ONH and periphery (Figure 12C). These relationships are complementary in that cells with larger breadth can contact more vessels than those with compressed morphology. Cells which contact more unique vessels are also more likely to contact smaller vessels, whereas cells with compressed morphology tend to contact larger vessels (Figures 12A–C).

## Discussion

Astrocytes are often described as stellate cells without distinguishing tissue location in the central nervous system (Schiweck et al., 2018). We demonstrate that this term does not accurately describe the majority of retinal astrocytes (Figures 1, 5) and recommend moving away from the term to describe them. We demonstrate that immunolabeling for pan-astrocyte markers such as GFAP and S100β underestimates a large degree of retinal astrocyte complexity (Figures 2, 3). We show that neither S100β nor GFAP can reveal all membrane protrusions, and their immunolabeling in isolation obscures the fact that most astrocytes contain large regions of planar membrane (Figures 2, 3). Additionally, GFAP content varies with retinal eccentricity (Figure 2) which complicates its use as a marker for cell density and proliferation. In our work, we used G-MORF mice (Supplementary Figures S1–S3) to reveal that individual astrocytes contain structural motifs which are predictive of the neuronal and vascular architecture of the inner retina (Figures 4–9, Table 1). These motifs help reduce the complexity of individual astrocytes by acting as building blocks from which the final cell shape can be constructed. The complexity revealed by V5 labeling is remarkable but not inconsistent with whole-cell dye filling in this paper (Figure 5) or those done by others (Luna et al., 2016). The strength of our study comes from the ability to visualize thousands of cells, and through this process recognize recurring motifs which in small sample sizes may appear random.

Recognition of these motifs invites questions regarding function. We identify that individual motifs contact distinct partners (axon, RGC soma, vasculature, or neighboring astrocytes; Figures 6–8, Supplementary Figure S4; Table 1). Beads and pads can contact RGC soma at the axon hillock region and on varicosities of RGC axons (Figure 7), two regions which are important for action potential generation and axon metabolism (Wang et al., 2003; Palmer and Stuart, 2006; Osborne, 2010). Due to their placement at these locations and the fact that each astrocyte contacts blood vessels, we speculate that these motifs are used to sample the ionic or metabolic state of RGCs, information which the cell could then use to influence vascular tone. Studies examining the functional interactions between astrocytes and axons largely focus on the synapse. Less is known about how they interact at Nodes of Ranvier and there are almost no studies which look at functional interactions along lengths of unmyelinated axon. However, this is precisely the research that is most applicable to the retina, where axon-astrocyte interactions occur at lengths of unmyelinated RGC axons. There has been work done in other regions of the CNS, namely with hippocampal CA3 pyramidal neurons. *In vitro* neuronal recordings in response to calcium uncaging in periaxonal astrocytes shows that astrocytes can directly modulate the shape of action potentials in these neurons and in doing so affect information transfer far from the synapse (Sasaki et al., 2011). Additionally, freeze-fracture electron micrographs of the primate retina have revealed electron rich regions of axon-glial connection (Black et al., 1984). These electron-rich regions of the axon showed high amounts of intramembranous particles on the E-face of the axolemma and were only present at the site of glial process abutment. The authors presumed these particles to be ion channels based on their morphological similarity to Nodes of Ranvier as well as immature axons prior to the establishment of myelin (Black et al., 1984). It is possible that these are regions of communication between retinal glia and RGC axons that serve to modulate the activity of both cells. Finally, beads are reminiscent of varicosities found on varicose projection astrocytes of the primate cerebral cortex whose function is unknown (Falcone et al., 2022). It is likely that determining the function of the bead motif in retinal astrocytes will inform the function of varicosities on these cerebral astrocytes.

Similarly, the placement of sails across bundles of axons and selectively across the surface of RGC soma suggests that they may be important for providing resources and exchanging metabolites across a larger area of neural retina than a single process could (Figure 6). They may also help maintain the integrity of axon bundles throughout life. Moreover, the structural distinction between tubes and end-feet signals that they have separate roles in the regulation of blood flow or the maintenance of the blood-retinal barrier. In the brain, laser ablation of astrocyte end-feet does not compromise the integrity of the blood–brain-barrier (Kubotera et al., 2019). In the retina, there may be functional divergence with end-feet controlling blood flow regulation and tube motifs participating in the blood-retinal barrier. Bristles make very fine connections to both neural structures and neighboring astrocytes (Figure 8). Like beads, bristles contact individual axons (Figures 8A–C) and likely play a role in sensing the ionic environment. We were most intrigued by astrocytes near the optic nerve head which use bristled processes to traverse multiple RGC axon bundles, making connections to individual axons in each (Figure 8B). Their morphology is strikingly similar to hippocampal dendritic spines, which can be lost in response to chronic stress (Bourne and Harris, 2008; Chen et al., 2008). While it is unlikely that these structures form synapses, the overall structure of the bristled process appears amenable to summing electrical information along its length for integration. This potentially allows a single astrocyte to sense the energetic demands of a large portion of retina relative to its own size. In glaucoma, the initial site of stress is the optic nerve head (Calkins, 2021; Wareham et al., 2022). It is possible that early in disease these bristles are damaged or retracted, presumably impairing blood flow regulation to a large patch of retina, leaving it susceptible to further insult. Bristles also trace individual axons as they emanate from cell bodies and fasciculate. We suspect that they help direct axons to their nearest bundle. We also observe bristles making connections to neighboring astrocytes, likely mediating communication within the network. In this paper, we refer to any process approximately 5 μm in length or shorter as a bristle, but we hypothesize that this classification will be refined due to the heterogeneity in shape we observe (Figure 8). Because each of these structural motifs appear to have distinct roles, we suspect that holes do as well. It is possible that they may serve as regions through which RGC axons protrude out into the NFL, similar to how cortical astrocytes are penetrated by axons and form holes to surround neuronal soma (Halassa et al., 2007; Torigoe et al., 2015).

During development, astrocytes and astrocyte progenitor cells migrate into the retina through the optic nerve head. Once through, they utilize the RGC axons as guides to direct their centrifugal migration to the periphery, establishing a network to direct angiogenesis and vascular development (O’Sullivan et al., 2017; Paisley and Kay, 2021). Their functional role as an intermediary between the RGCs and the vasculature continues into adulthood where they help form NVUs and regulate blood flow (Takano et al., 2006; Newman, 2015; Wareham and Calkins, 2020). Our work highlights this intimate relationship, revealing that every retinal astrocyte contacts both neuronal and vascular elements (Figure 9). In most cases, connections are made to RGC axons as well as soma. Furthermore, it is most common for connections to be made to multiple unique blood vessels. These data demonstrate a greater degree of NVU participation than was previously known, opening the possibility that blood flow is tightly controlled at the spatial level of a single astrocyte domain. We also describe classes of connections made between astrocytes and the vasculature (Figure 10). These classes form a lateralization in our UMAP embedding, indicative of underlying structure (Figure 10). Importantly, we demonstrate that cells making enveloping connections are distinct from pericytes through their expression of GFAP and lack of NG2 expression (Figure 11). NG2 is a common marker for pericytes, and it is well reported that pericytes are GFAP-negative (Dore-Duffy et al., 2006; Trost et al., 2016; Bruckner et al., 2018; Mai-Morente et al., 2021). Additionally, cells making enveloping connections were found exclusively in the NFL and not in deeper layers of the retina where pericytes are also present (Bruckner et al., 2018). Interestingly, they appear to envelop not only the vasculature but also NG2-positive pericytes.

Astrocyte morphology also varies with distance from the ONH (Figure 12). In the periphery and near the nerve head, astrocytes are compressed relative to those in the mid-retina. This is coincident with an increase in the percentage of projection area which is positive for GFAP (Figures 2F, 12). We speculate that these differences are due to the biomechanical stresses which occur near the ora serrata and ONH as well as the non-homogenous stiffness profile of the inner retina (Sigal et al., 2004; Franze et al., 2011; Fazio et al., 2018; Ma et al., 2019). Atomic force microscopy shows that retinal stiffness in guinea pigs is relatively low at the ONH and periphery relative to the mid-retina (Franze et al., 2011). Because the two sites of firm attachment for the retina are the ora serrata and ONH, the posteriorly directed force of intraocular pressure at the ONH likely also increases the force experienced in the periphery. Astrocyte shape compression in these regions could be in response to elevated forces felt in their microenvironment. These populations may be important in glaucoma, where elevated intraocular pressure acts at the optic nerve head to deform the structure posteriorly (Ma et al., 2019). If this shape compression is inducible by force or injury, it may contribute to observations of reactive astrocytes with elevated GFAP and process hypertrophy (Fernández-Sánchez et al., 2015; Lambert et al., 2019; Holden et al., 2022; Rutigliani et al., 2022). Moreover, we connect this shape compression to blood flow by observing that these compressed cells are less likely to contact multiple unique vessels as are their counterparts in the mid-retina. It is possible that blood flow regulation in these regions is less refined than in the mid-retina, rendering the tissue more vulnerable to damage.

Single-cell RNA sequencing currently drives our understanding of astrocytes in both naïve and disease contexts. It has revealed a heterogeneous response to injury and disease, suggesting cells exist on a reactivity spectrum between neurotoxic (A1) and neuroprotective (A2) phenotypes (Escartin et al., 2021; Hasel et al., 2021; Debom et al., 2022). Due to the diversity in molecular states identified, it has been proposed that classification of reactivity should include not only transcriptomic, proteomic, and functional criteria, but also morphology (Escartin et al., 2021). Our work proposes a method and feature set to describe retinal astrocyte morphology with this goal in mind and supports a spectral view of the population on a morphological basis. While focusing on single-cell transcriptomic profiles, there has been less exploration of classification based on single-cell morphology (Liddelow and Barres, 2015; Wheeler et al., 2020; Hasel et al., 2021). The studies which do focus on morphology are centered on the optic nerve head and optic nerve proper rather than the retina (Sun et al., 2010; Lye-Barthel et al., 2013). Our work demonstrates the utility of morphological classification in the retina, discovering functionally relevant structural motifs (Figures 4–8). Our results imply that while retinal astrocyte morphology is indeed heterogeneous, it is not random. Instead, it appears to be almost entirely dependent on a given cell’s local environment (Figures 4–12). Each individual region of the retina is subject to different biomechanical forces and has a distinct fingerprint of vessels and neuronal architecture. Thus, it seems logical that every astrocyte would also have a unique structure. We show that the structure of individual astrocytes can be quantified in a systematic way (Figures 1, 2, 9–12 and Table 1), thereby opening the possibility of quantifying small changes which occur during the progression of disease. Following induction of gliosis, astrocytes alter the number and cytoskeletal composition of their processes (Pekny and Nilsson, 2005; Burda and Sofroniew, 2014; Escartin et al., 2021). We suspect that these observations encompass alterations to structural motifs with consequences for cell function. If these motifs play a role in neuronal support and vascular regulation, their state change could contribute to or incite pathogenic processes. Individual astrocytes have the potential to functionally alter their microenvironment, and by extension, their resident tissue. G-MORF mice will enable us to understand these changes and how they contribute to disease processes in the CNS.

## Data availability statement

The raw data supporting the conclusions of this article will be made available by the authors, without undue reservation.

## Ethics statement

The animal studies were approved by Vanderbilt Animal Care and Use Program. The studies were conducted in accordance with the local legislation and institutional requirements. Written informed consent was obtained from the owners for the participation of their animals in this study.

## Author contributions

DC and LW provided resources, supervision, project management, and manuscript editing. JH conceptualized the study, wrote the software, conducted the experiments, analyzed the data, and wrote the manuscript. All authors contributed to the article and approved the submitted version.

## Funding

Research was funded in part by an unrestricted grant to DC from Research to Prevent Blindness, The Potocsnak Family Vision Research Center, P30 EY008126 and U24 grant EY029903 for imaging. Experiments were performed in part through the use of the Vanderbilt Cell Imaging Shared Resource (supported by NIH grants CA68485, DK20593, DK58404, DK59637, and EY08126).

## Conflict of interest

The authors declare that the research was conducted in the absence of any commercial or financial relationships that could be construed as a potential conflict of interest.

## Publisher’s note

All claims expressed in this article are solely those of the authors and do not necessarily represent those of their affiliated organizations, or those of the publisher, the editors and the reviewers. Any product that may be evaluated in this article, or claim that may be made by its manufacturer, is not guaranteed or endorsed by the publisher.
