# Supplementary material for: Retinal astrocyte morphology predicts integration of vascular and neuronal architecture
Source: Front Neurosci. 2023 Aug 9;17:1244679. doi: 10.3389/fnins.2023.1244679 (PMC10445659; doi:10.3389/fnins.2023.1244679)
Supplement: Supplementary file 1 [file Data_Sheet_1.docx]

Supplementary Material

Retinal Astrocyte Morphology Predicts Integration of Vascular and Neuronal Architecture

Joseph M. Holden^1,2^, Lauren K. Wareham^1^, David J. Calkins^1*^

^1^Department of Ophthalmology and Visual Sciences, Vanderbilt University Medical Center, Nashville, TN 37212

^2^Vanderbilt Neuroscience Graduate Program, Vanderbilt University, Nashville, TN 37212

*** Correspondence:**David J. Calkins
[david.j.calkins@vumc.org](mailto:david.j.calkins@vumc.org)


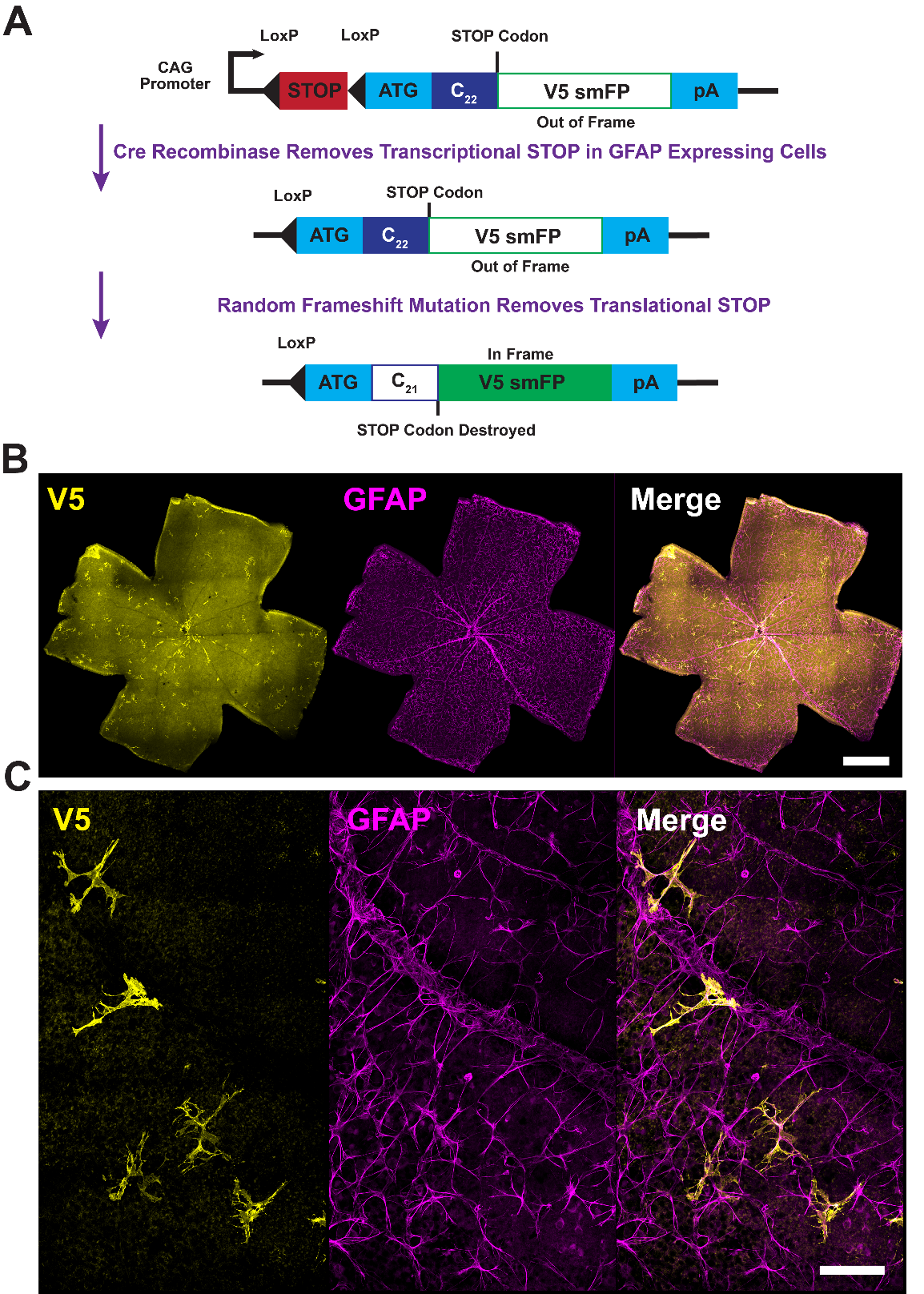


**Fig. S1: G-MORF Mouse Model (A)** The key aspects of the G-MORF genetics are both its transcriptional and translational STOPs, which must be removed before expression of the spaghetti monster fluorescent protein. This is accomplished through a combination of random mutation and Cre recombinase expression. This mouse is a cross between MORF3 and GFAP Cre 77.6 **(B)** V5 labeling in G-MORF mice is sparse and largely well-separated. Shown is an entire wholemount retina labeled for V5 and GFAP (scale 1mm) **(C)** Five GFAP-positive, well-separated, single retinal astrocytes with full membranous morphology (scale 100 µm)


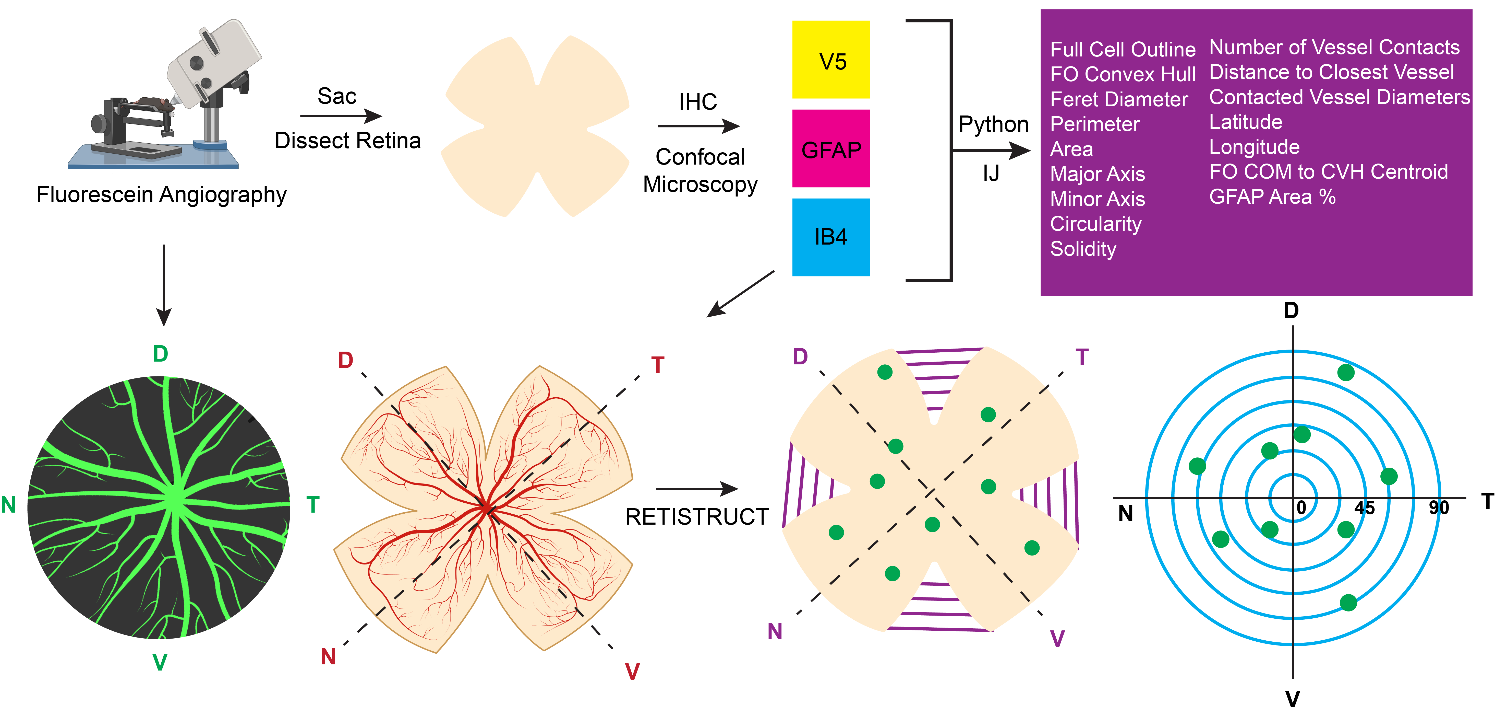


**Fig. S2: Methods** Prior to sacrificing mice, fluorescein angiography was performed to get a fingerprint of the retinal vasculature. This was used to determine orientation in IB4-labeled retinas. Retinas were dissected and labeled for V5, GFAP, and IB4. Custom Python scripts and ImageJ macros were used to quantify a variety of morphological parameters. Individual cells’ positions were noted in V5-labeled, montage retinal images. These coordinates were fed into RETISTRUCT which maps the wholemount retina to a hemisphere surface to acquire geographic coordinates of each cell in native retinal space.


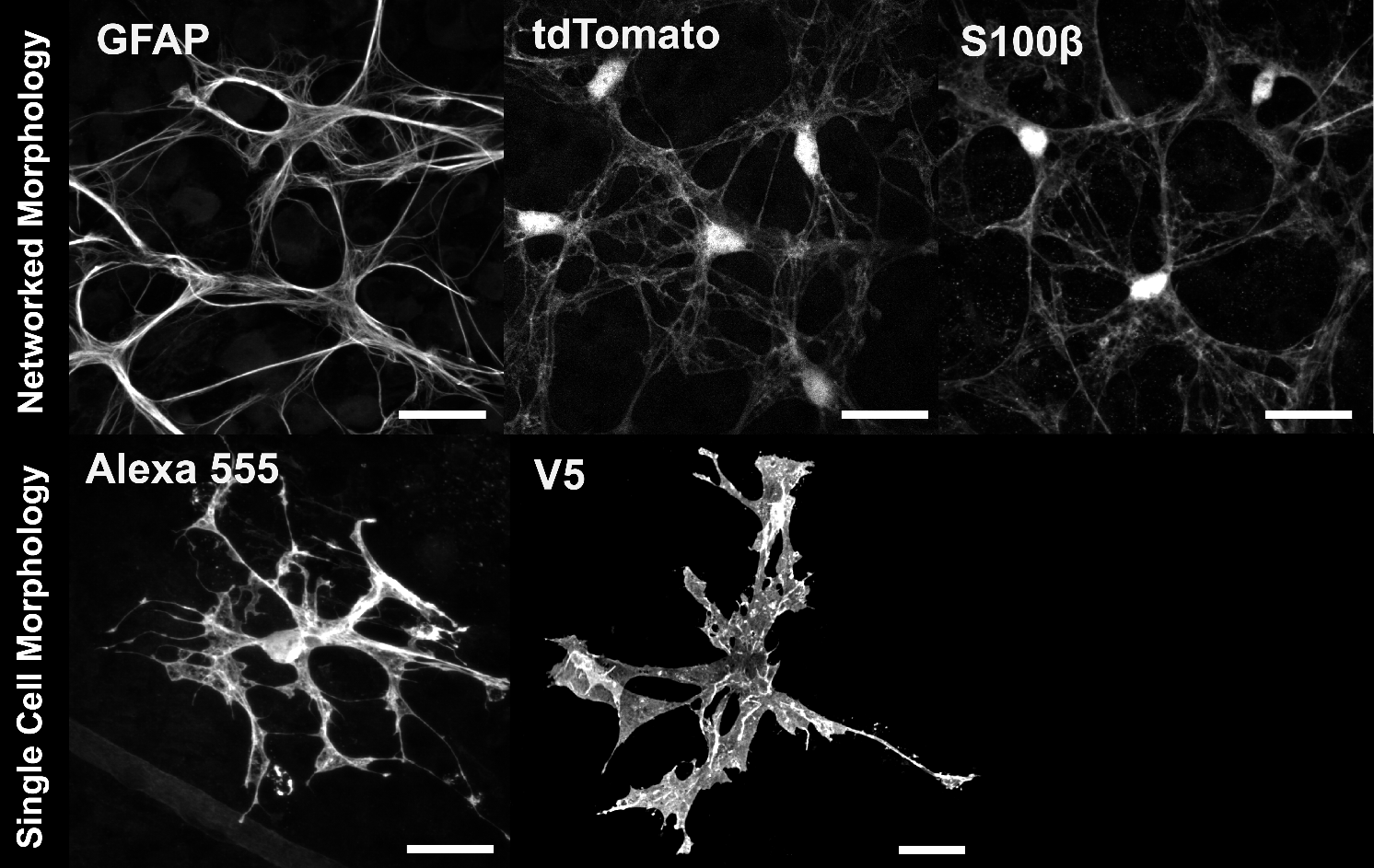


**Fig. S3: V5 Labeling in G-MORF Retinas Allows for Detailed Visualization of Astrocytes.** Images demonstrate the ability of various methods of labeling to visualize astrocyte morphology. From left to right, top to bottom images show GFAP, GFAP-controlled Cre-dependent cytosolic tdTomato expression from a CAG promoter, S100β, whole-cell dye filling with Alexa 555, and V5 labeling in G-MORF mice. Adding to the limitations outlined in **Fig. 2**, labeling for GFAP lacks the ability to visualize cell membranes for accurate morphology. Additionally, because astrocytes form an extensive network, distinguishing individual cells with high confidence is not possible when used in isolation. Labeling astrocyte-specific cytosolic proteins such as endogenous S100β or transgene products such as tdTomato captures some but not all membranous morphology. Like cytoskeletal labeling, it also lacks the ability to distinguish individual cells. Whole-cell patch combined with intracellular dye filling is limited by technical difficulty and throughput but reveals individual cell morphology. V5 labeling in G-MORF mice has the advantages of immunolabeling and whole-cell dye filling without their limitations. Scale Bars indicate 25µm.


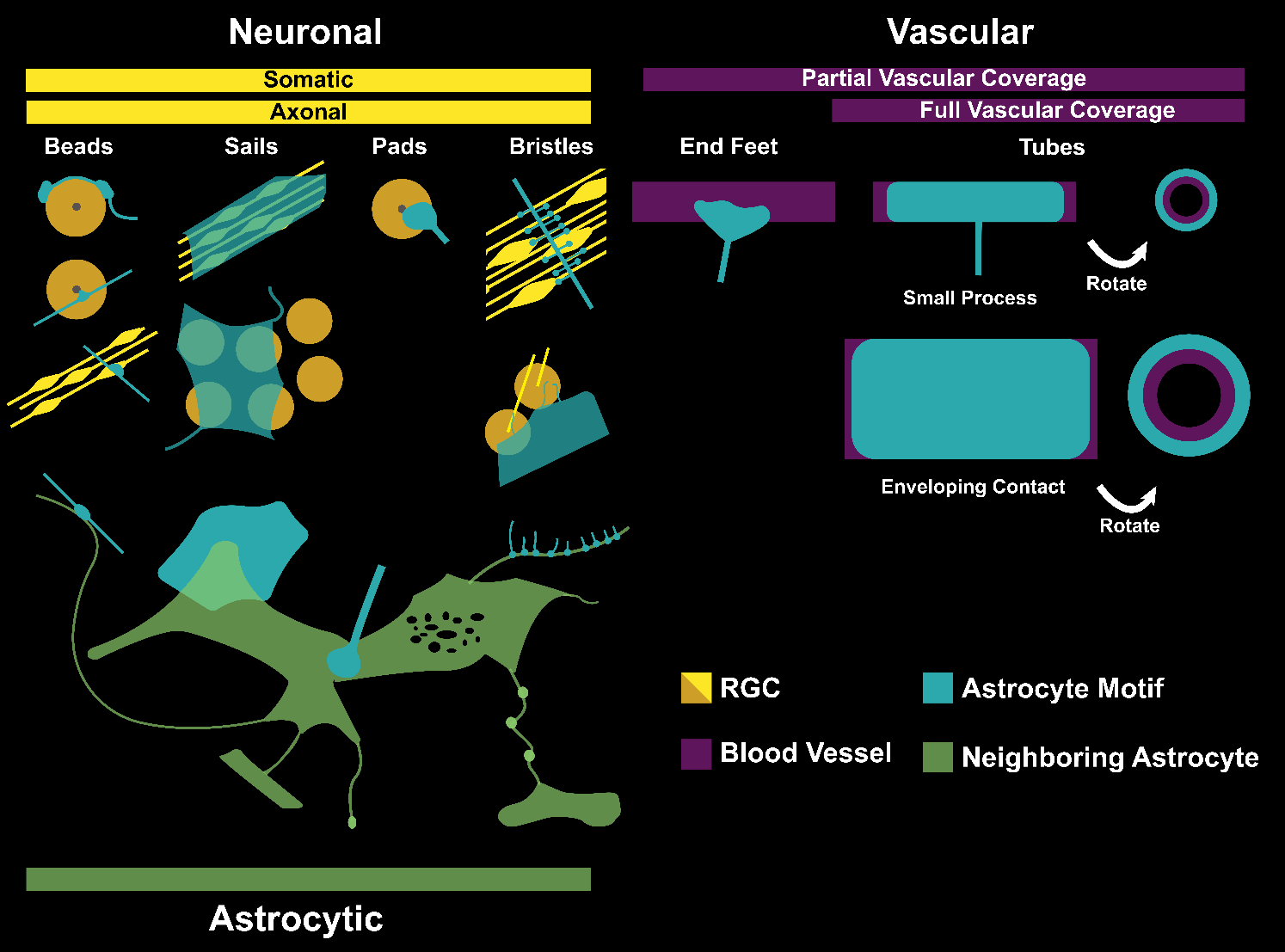


**Fig. S4:** This schematic shows the major astrocyte structural motifs which have defined partner interactions. Beads, sails, pads, and bristles interact with neuronal and neighboring astrocytic elements whereas end-feet and tubes contact the vasculature.
